# Neuropathological consensus criteria for the evaluation of Lewy pathology in post-mortem brains: a multi-centre study

**DOI:** 10.1007/s00401-020-02255-2

**Published:** 2021-01-05

**Authors:** Johannes Attems, Jon B. Toledo, Lauren Walker, Ellen Gelpi, Steve Gentleman, Glenda Halliday, Tibor Hortobagyi, Kurt Jellinger, Gabor G. Kovacs, Edward B. Lee, Seth Love, Kirsty E. McAleese, Peter T. Nelson, Manuela Neumann, Laura Parkkinen, Tuomo Polvikoski, Beata Sikorska, Colin Smith, Lea Tenenholz Grinberg, Dietmar R. Thal, John Q. Trojanowski, Ian G. McKeith

**Affiliations:** 1 Translational and Clinical Research Institute, Newcastle University, Campus for Ageing and Vitality, Newcastle upon Tyne, UK; 2 Department of Neurology, Fixel Center for Neurological Diseases, University of Florida, Gainesville, USA; 3 Department of Pathology and Laboratory Medicine, Center for Neurodegenerative Disease Research, Perelman School of Medicine at the University of Pennsylvania, Philadelphia, PA USA; 4 Division of Neuropathology and Neurochemistry, Department of Neurology, Medical University of Vienna, Vienna, Austria; 5 Neurological Tissue Bank of the Biobank-Hospital Clinic-IDIBAPS, Barcelona, Spain; 6 Department of Brain Sciences, Imperial College London, London, UK; 7 Brain and Mind Centre and Faculty of Medicine and Health, School of Medical Sciences, University of Sydney, Sydney, Australia; 8 Institute of Pathology, Faculty of Medicine, University of Szeged, Szeged, Hungary; 9 Department of Old Age Psychiatry, Institute of Psychiatry Psychology and Neuroscience, King’s College London, London, UK; 10 Centre for Age-Related Medicine, SESAM, Stavanger University Hospital, Stavanger, Norway; 11 Department of Neurology, ELKH-DE Cerebrovascular and Neurodegenerative Research Group, University of Debrecen, Debrecen, Hungary; 12 Institute of Clinical Neurobiology, Vienna, Austria; 13 Department of Laboratory Medicine and Pathobiology and Tanz Centre for Research in Neurodegenerative Disease, University of Toronto, Toronto, ON Canada; 14 Laboratory Medicine Program, University Health Network, Toronto, ON Canada; 15 South West Dementia Brain Bank and Institute of Clinical Neurosciences, School of Clinical Sciences, University of Bristol, Bristol, UK; 16 Department of Pathology and Sanders-Brown Center On Aging, University of Kentucky, Lexington, KY USA; 17 Department of Neuropathology, University Hospital of Tübingen, Tübingen, Germany; 18 Molecular Neuropathology of Neurodegenerative Diseases, German Center for Neurodegenerative Diseases, Tübingen, Germany; 19 Oxford Parkinson’s Disease Centre, University of Oxford, Oxford, UK; 20 Nuffield Department of Clinical Neurosciences, University of Oxford, Oxford, UK; 21 Department of Molecular Pathology and Neuropathology, Medical University of Lodz, Lodz, Poland; 22 Centre for Clinical Brain Sciences, University of Edinburgh, Edinburgh, UK; 23 Departments of Neurology and Pathology, University of California San Francisco, San Francisco, USA; 24 Department of Pathology, University of Sao Paulo, Sao Paulo, Brazil; 25 Laboratory of Neuropathology, Department of Pathology, Department of Imaging and Pathology, and Leuven Brain Institute, KU Leuven, UZ Leuven, Leuven, Belgium

**Keywords:** Lewy body disease, Diagnostic neuropathology

## Abstract

Currently, the neuropathological diagnosis of Lewy body disease (LBD) may be stated according to several staging systems, which include the Braak Lewy body stages (Braak), the consensus criteria by McKeith and colleagues (McKeith), the modified McKeith system by Leverenz and colleagues (Leverenz), and the Unified Staging System by Beach and colleagues (Beach). All of these systems use semi-quantitative scoring (4- or 5-tier scales) of Lewy pathology (LP; *i.e.*, Lewy bodies and Lewy neurites) in defined cortical and subcortical areas. While these systems are widely used, some suffer from low inter-rater reliability and/or an inability to unequivocally classify all cases with LP. To address these limitations, we devised a new system, the LP consensus criteria (LPC), which is based on the McKeith system, but applies a dichotomous approach for the scoring of LP (*i.e.*, “absent” vs. “present”) and includes amygdala-predominant and olfactory-only stages. α-Synuclein-stained slides from brainstem, limbic system, neocortex, and olfactory bulb from a total of 34 cases with LP provided by the Newcastle Brain Tissue Resource (NBTR) and the University of Pennsylvania brain bank (UPBB) were scanned and assessed by 16 raters, who provided diagnostic categories for each case according to Braak, McKeith, Leverenz, Beach, and LPC systems. In addition, using LP scores available from neuropathological reports of LP cases from UPBB (*n* = 202) and NBTR (*n* = 134), JT (UPBB) and JA (NBTR) assigned categories according to all staging systems to these cases. McKeith, Leverenz, and LPC systems reached good (Krippendorff’s *α* ≈ 0.6), while both Braak and Beach systems had lower (Krippendorff’s *α* ≈ 0.4) inter-rater reliability, respectively. Using the LPC system, all cases could be unequivocally classified by the majority of raters, which was also seen for 97.1% when the Beach system was used. However, a considerable proportion of cases could not be classified when using Leverenz (11.8%), McKeith (26.5%), or Braak (29.4%) systems. The category of neocortical LP according to the LPC system was associated with a 5.9 OR (*p* < 0.0001) of dementia in the 134 NBTR cases and a 3.14 OR (*p* = 0.0001) in the 202 UPBB cases. We established that the LPC system has good reproducibility and allows classification of all cases into distinct categories. We expect that it will be reliable and useful in routine diagnostic practice and, therefore, suggest that it should be the standard future approach for the basic post-mortem evaluation of LP.

**Supplementary Information:**

The online version contains supplementary material available at 10.1007/s00401-020-02255-2.

## Introduction

Lewy body disease (LBD) encompasses Parkinson’s disease (PD), PD with mild cognitive impairment (PD-MCI), PD with dementia (PDD), and dementia with Lewy bodies (DLB), which all have a characteristic clinical presentation and associated clinical diagnostic criteria [10, 15, 18, 23]. The neuropathological hallmark of these clinically defined conditions is Lewy pathology (LP), which encompasses α-synuclein aggregates in nerve cell bodies and processes: Lewy bodies (LB) and Lewy neurites (LN), respectively. However, LP may also be seen in individuals lacking distinct clinical symptoms. The term incidental LBD was initially coined for individuals who lacked Parkinsonian or cognitive symptoms but had minimal LP restricted to the brainstem, but more recently, it has been expanded to encompass amygdala-predominant and olfactory-only LP [2, 3, 13].

The heterogeneity of LP is a challenge for neuropathological classification systems. Diagnostic categories must reflect the wide range of LP severity and anatomical distribution, while also enabling robust inter-rater reliability. The existing neuropathological classification systems used for the diagnosis and staging of LP include the Braak LB stages (Braak) [5], the DLB consensus criteria published by McKeith and colleagues (McKeith) [17], the modified DLB consensus criteria by Leverenz and colleagues (Leverenz) [14], and the Unified Staging System for LBD by Beach and colleagues (Beach) [3]. These staging systems are based on the semi-quantitative scoring of LBs and LNs in neuroanatomically defined regions, in particular the dorsal motor nucleus of the vagal nerve, locus coeruleus, substantia nigra, transentorhinal cortex, amygdala, cingulate cortex, temporal cortex, frontal cortex, and parietal cortex. For the McKeith, Leverenz, and Beach systems the severity of LBs and LNs is scored on a 5-tier scale: 0 = absent, 1 = sparse LBs or LNs, 2 = more than one LB per high power field and sparse LNs, 3 = more than four LBs and scattered LNs in a low power field, 4 = numerous LBs and LNs, as illustrated by McKeith and colleagues [17]. For the Braak system, a four-tier scale is used to reflect the extent of α-synuclein immunolabelling: 0 = absent, 1 = “slight”, 2 = “moderate”, 3 = “severe”, as described by Braak and colleagues [5].

The BrainNet Europe Consortium (BNE) found mean inter-rater agreement rates of 65% (range 32–100%) for the Braak system and 81% (range 45–100%) for the McKeith system when 22 experts assessed 31 cases which all showed some LB pathology [2]. BNE developed a new protocol which was not based on semi-quantitative scoring but simply on the presence or absence of LBs and/or LNs, and added the category “amygdala predominant” for cases with pathology most severe in the amygdala and less pronounced in brainstem areas. This protocol achieved inter-rater agreement of 83% for the Braak system and 84% for the McKeith system [2]. Similarly, Müller and colleagues applied the Braak system in an inter-rater study where a semi-quantitative score was only needed for stage 6, while stages 1–5 could be assigned based on the presence of LP in the relevant areas and achieved an inter-rater reliability of at least 76% [20].

While all of these neuropathological staging systems are widely used, they exhibit relatively low inter-rater reliability and frequently make cases diagnostically unclassifiable; *e.g.,* a case with severe LP in the neocortex but only mild in the brainstem cannot be classified in the Braak system and when using the McKeith system cases may sometimes be assigned to more than one category. Hence, there is a need for a LP staging system that shows high inter-rater reliability, allows for the unequivocal classification of all possible cases, and is readily applicable in neuropathological routine diagnostics. To address this unmet need, we developed a new LP classification system based on a modification of the McKeith system and which uses the dichotomized approach introduced by the BNE. 16 raters in 13 different centres used this new classification system as well as the Braak, McKeith, Leverenz, and Beach systems to score and stage LP in 34 cases. In addition, regional LP scores retrieved from diagnostic neuropathological reports from the University of Pennsylvania brain bank (UPBB) and the Newcastle Brain Tissue Resource (NBTR) were used to re-assign LP categories according to all systems for 363 LP cases.

## Materials and methods

### Classification system

**Fig. 1:**
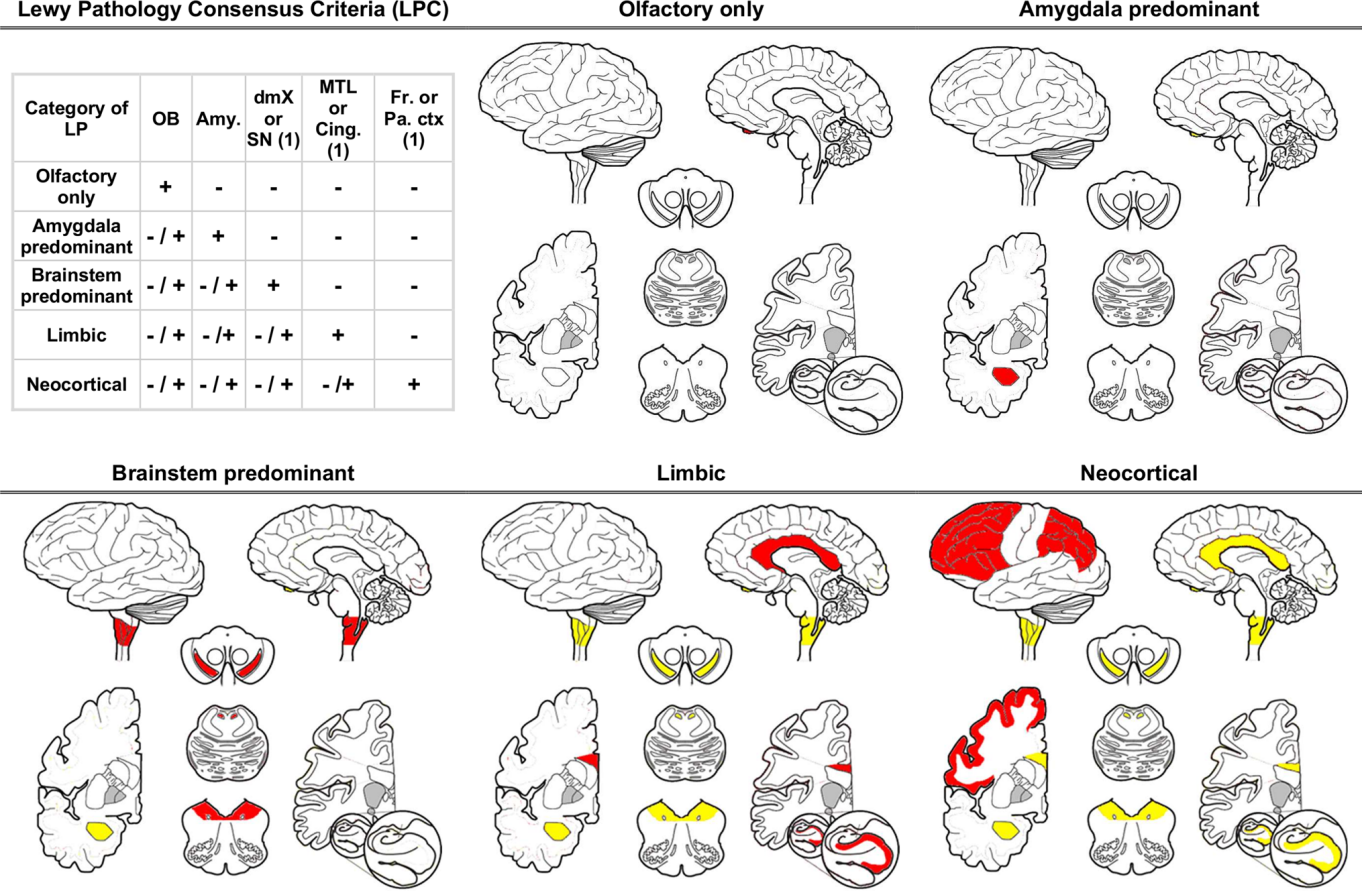
The new Lewy pathology consensus criteria (LPC). Yellow colour, LP can be absent ( −) or present ( +); red colour, LP must be present ( +). Of note: while presence ( +) of LP in the amygdala and in medial–temporal lobe or cingulate cortex is not mandatory for assigning a category of limbic and neocortical LP, respectively, we emphasise that it is highly unlikely that LP will be absent ( −) in the amygdala of limbic LP and in the medial–temporal lobe or cingulate cortex of neocortical LP. *LP* Lewy-related pathology; *OB* olfactory bulb/tract; *dmX* dorsal motor nucleus of vagal nerve/ medulla; *SN* substantia nigra; *Amy* amygdala; *MTL* medial–temporal cortex; *Cing* cingulate cortex; *Fr*. or Pa. ctx, frontal or parietal cortex

**Fig. 2:**
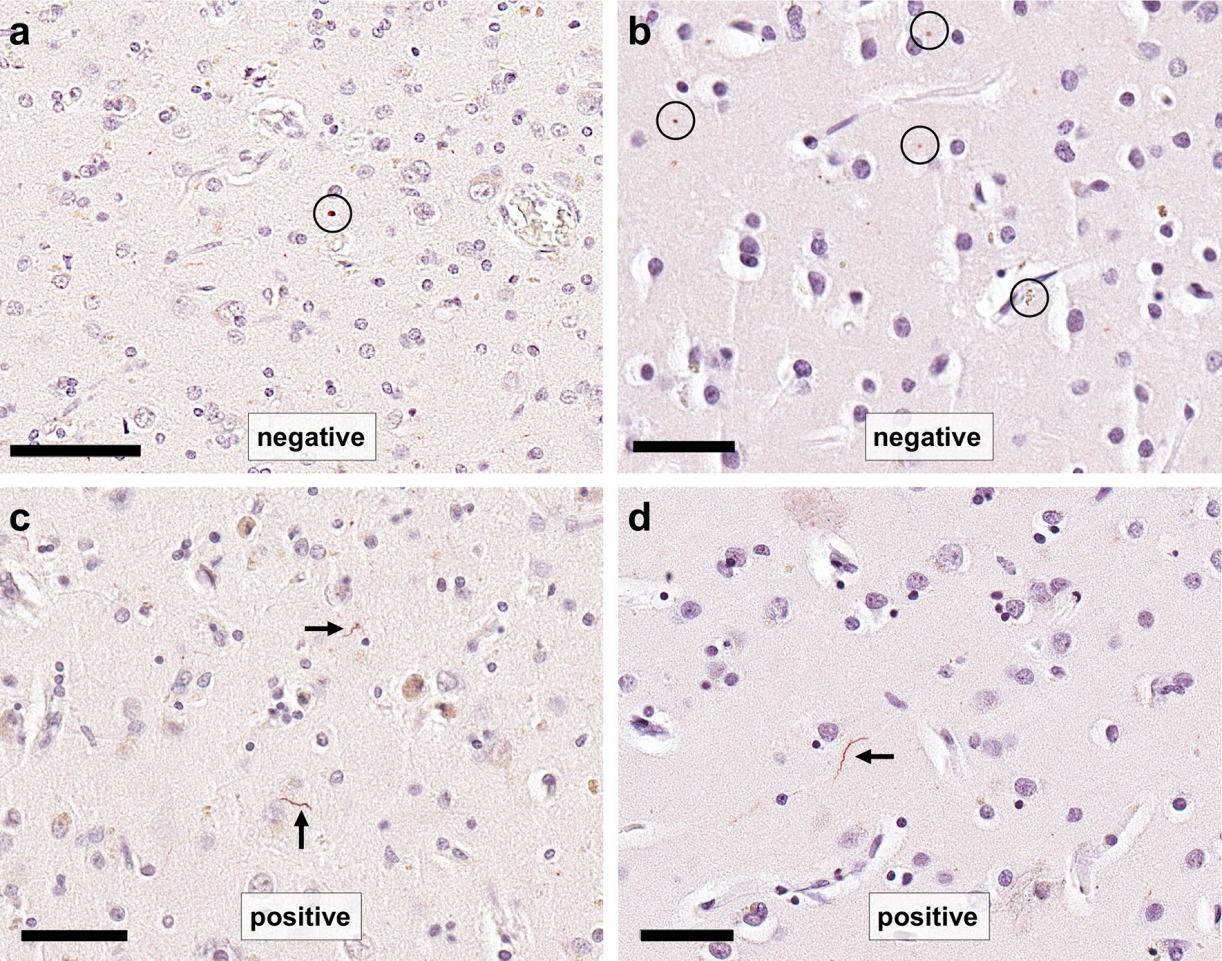
Photomicrographs of α-synuclein stained slides showing dot like, artefactual positivity that should not be considered positive for scoring (encircled in **a** and **b**) and single α-synuclein-positive Lewy neurites (arrows in **c** and **d**) that would yield a score of “positive”. Scale bar in a: 70 μm, in b, c, and d: 50 μm

Our proposed new neuropathological classification system for LP, the LP consensus criteria (LPC), is based on dichotomized scoring of LB or LN, *i.e.* as present ( +) or absent ( −), in olfactory bulb, dorsal motor nucleus of the vagal nerve (dmX), substantia nigra, amygdala, cingulate cortex, medial–temporal cortex, frontal cortex, and parietal cortex (Fig. 1). A region is scored positive if the severity of LB or LN would be at least scored 1 (sparse LBs or LNs) according to the McKeith system (Fig. 2) [17]. The possible diagnostic categories are olfactory-only, amygdala-predominant, brainstem, limbic, and neocortical LP as suggested in the Fourth Consensus Report of the DLB Consortium [18]. Of note, all regions need to be assessed, but for the diagnosis of brainstem, limbic, and neocortical LP not all regions that are characteristic for the respective LP group need to be positive: *e.g.*, a case with a LB or LN score of 1 in either frontal or parietal cortex would be classified as neocortical LP (Fig. 1).

### Neuropathological samples

Human post-mortem brain tissue for the multi-rater assessment included 34 cases showing varying degrees of LP was obtained from the NBTR, (*n* = 13), with the approval of the joint Ethics Committee of Newcastle and North Tyneside Health Authority and in accordance with NBTR brain banking procedures, and from the UPBB (*n* = 21). None of the cases had any indication for a genetic synucleinopathy.

At NBTR, the right hemisphere, brainstem, and cerebellum were immersion-fixed in 4% aqueous formalin for 4–6 weeks. Routine tissue blocks were dissected for neuropathological diagnosis. The blocks were processed through increasing concentrations of alcohol and chloroform before being embedded in paraffin wax. Sections were cut at 6 μm. Those for immunohistochemistry underwent antigen retrieval and were incubated with antibody to α-synuclein (KM51 clone, 1:200. Leica, UK), which detects full length α-synuclein. Pathological protein aggregates were visualised using the Menarini X-Cell-Plus HRP Detection Kit (Menarini, Berkshire, UK), with 3,3′-diaminobenzidine as the chromogen.

At UPBB tissue was fixed in 10% neutral buffered formalin for one set of blocks, and 70% ethanol with 150 mM NaCl for another set of blocks (for details see [29]). One hemisphere was cut coronally at 1–1.5 cm intervals and cortical and subcortical blocks were taken. The brainstem was cut perpendicular to the neuraxis and cerebellum parasagittal at 1 cm intervals. The day after the autopsy, the tissue blocks were placed in cassettes and they are embedded in paraffin wax and cut at 6–10 μm for histology. Syn303 (mAb, 1:16,000, generated in the CNDR) was used to detect the presence of pathological α-synuclein (epitopes with amino acid residues 2–4). Bound primary antibody was visualized by the avidin–biotin detection method (VECTASTAIN ABC kit; Vector Laboratories, Burlingame, CA) with ImmPACT diaminobenzidine peroxidase substrate (Vector Laboratories) as the chromogen [29].

### Multi-rater assessment

Immunohistochemical sections (α-synuclein) that included dmX (medulla section), substantia nigra, amygdala, cingulate gyrus, medial-temporal cortex (parahippocampal gyrus), frontal cortex and parietal cortex from all 34 cases and from 13 olfactory bulbs (NBTR cases) were scanned using a Leica SCN 400 scanner at 40 × magnification (Supplementary Table 1, online resource). The scanned images, which included the entire section, were uploaded to a server and assessed by 16 raters (BS, DRT, EG, GH, GK, JA, JBT, JQT/EBL, KEM, LP, LTG, LW, MN, SL, TH, and TP); the Leica software (Aperio ImageScope, version 11.2) allowed for virtual slide navigation across the entire section and magnification comparable to a 40 × objective on a microscope (approx. 400 × magnification), so that even small neurites could be detected. The raters were blinded to any clinical or neuropathological diagnosis and by following the respective scoring and staging guidelines [3, 5, 14, 17], they assigned each case to a category within the Braak, McKeith, Leverenz, Beach, and LPC systems. In addition, raters’ scores were used to assign categories according to the dichotomized method suggested by BrainNet Europe for Braak and McKeith systems [2].

### Re-classification of archival cases

202 cases from UPBB and 134 cases from NBTR were assigned to a category according to Braak, McKeith, Leverenz, and LPC systems, using the semi-quantitative scores already available from the initial diagnostic assessment. The assignment was performed blinded to the original diagnoses by JBT for UPBB cases and JA for NBTR cases. Of note, none of the cases was initially diagnosed with a genetic synucleinopathy.

### Statistical analysis

The median was used as a measure of central tendency and the 25th and 75th percentiles to evaluate variability. We used Krippendorff’s α, as opposed to Cohen’s *κ* which is often used in multi-site assessments (*e.g.,* for NIA-AA guidelines [19]), because the former allows for missing data (non-classifiable cases, were not considered to have an assignable stage) and, like Fleiss’ kappa, is capable of including multiple raters in a single statistic. We did not use weighted statistics as we did not assume that there is a single order for ordering the categories. Therefore, all the possible differences in staging assignments between raters had the same weight/impact on the score. Logistic regression was used to assess the odds of a dementia diagnosis at the time of death when cases were subdivided according to limbic versus neocortical LPC stages, after adjusting for the Braak neurofibrillary tangle stage (V/VI versus lower stages).

## Results

### Inter-rater evaluation of staging systems

Supplementary Figs. 1–9 (online resource) show the semi-quantitative scores assigned to each area of the 34 cases. Overall, inter-rater reliability was moderate to high, with lowest reliability for the amygdala, medulla and olfactory bulb sections (Fig. 3a).

**Fig. 3:**
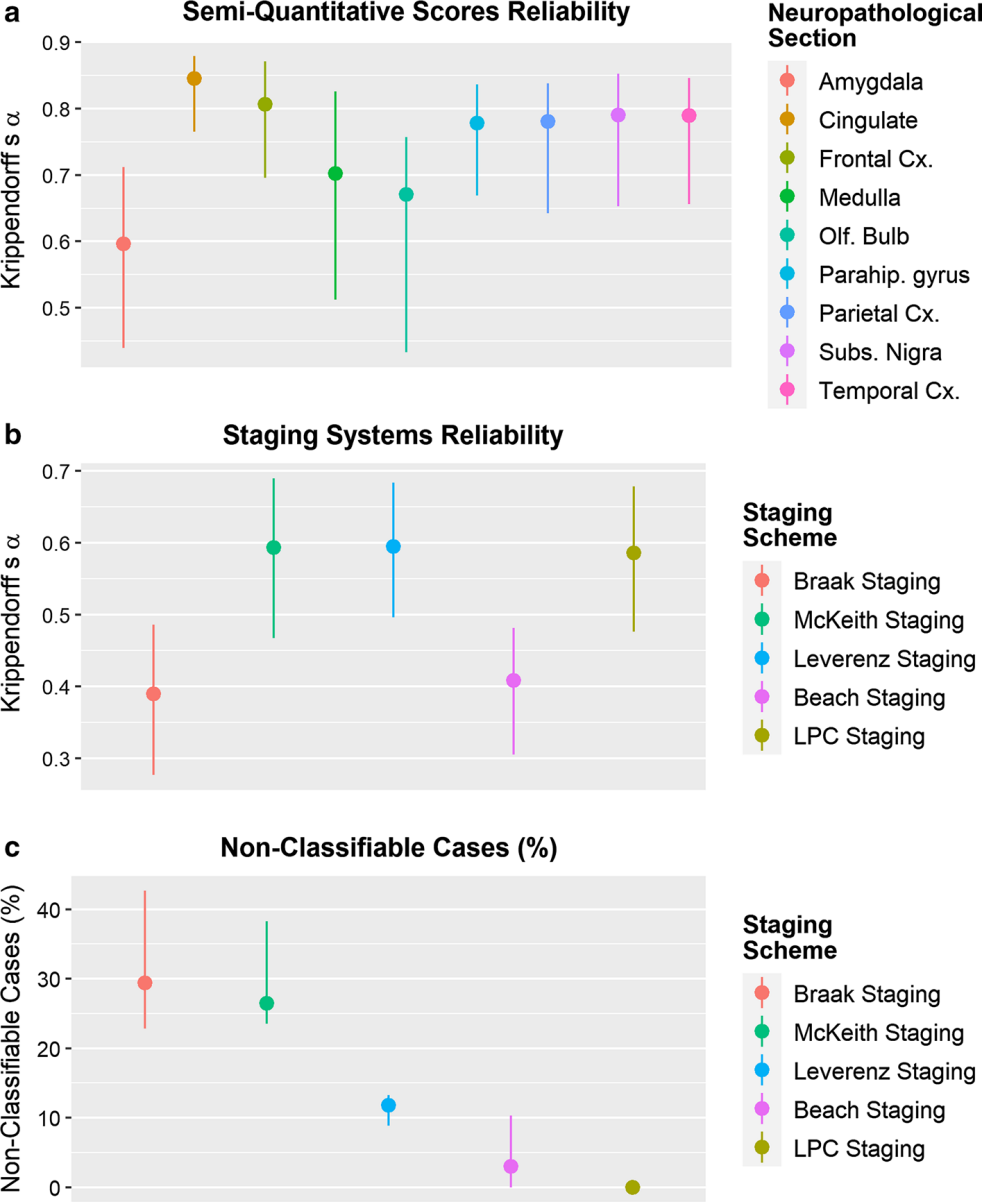
Inter-rater reliability (Krippendorff’s α) for semi-quantitative scores (**a**) and diagnostic categories assigned using the different staging systems (**b**). Percentages of cases that were deemed non-classifiable by the majority of raters (median and 25th and 75% percentiles) (**c**)

Based on their semi-quantitative scores raters classified each case according to the five staging systems. Overall, McKeith (Krippendorff’s *α*: 0.59), Leverenz (Krippendorff’s α: 0.59), and LPC (Krippendorff’s *α*: 0.59) systems reached good inter-rater reliability, whereas Braak (Krippendorff’s *α*: 0.39) and Beach (Krippendorff’s *α*: 0.41) systems had lower reliability (Fig. 3b). There were considerable differences between the staging systems in the percentage of cases that were not assigned any stage and, therefore, deemed non-classifiable by the majority of raters, with LPC (0%), and Beach (2.9%) systems performing best, followed by Leverenz (11.8%), McKeith (26.5%) and Braak (29.4%) systems (Fig. 3c).

**Fig. 4:**
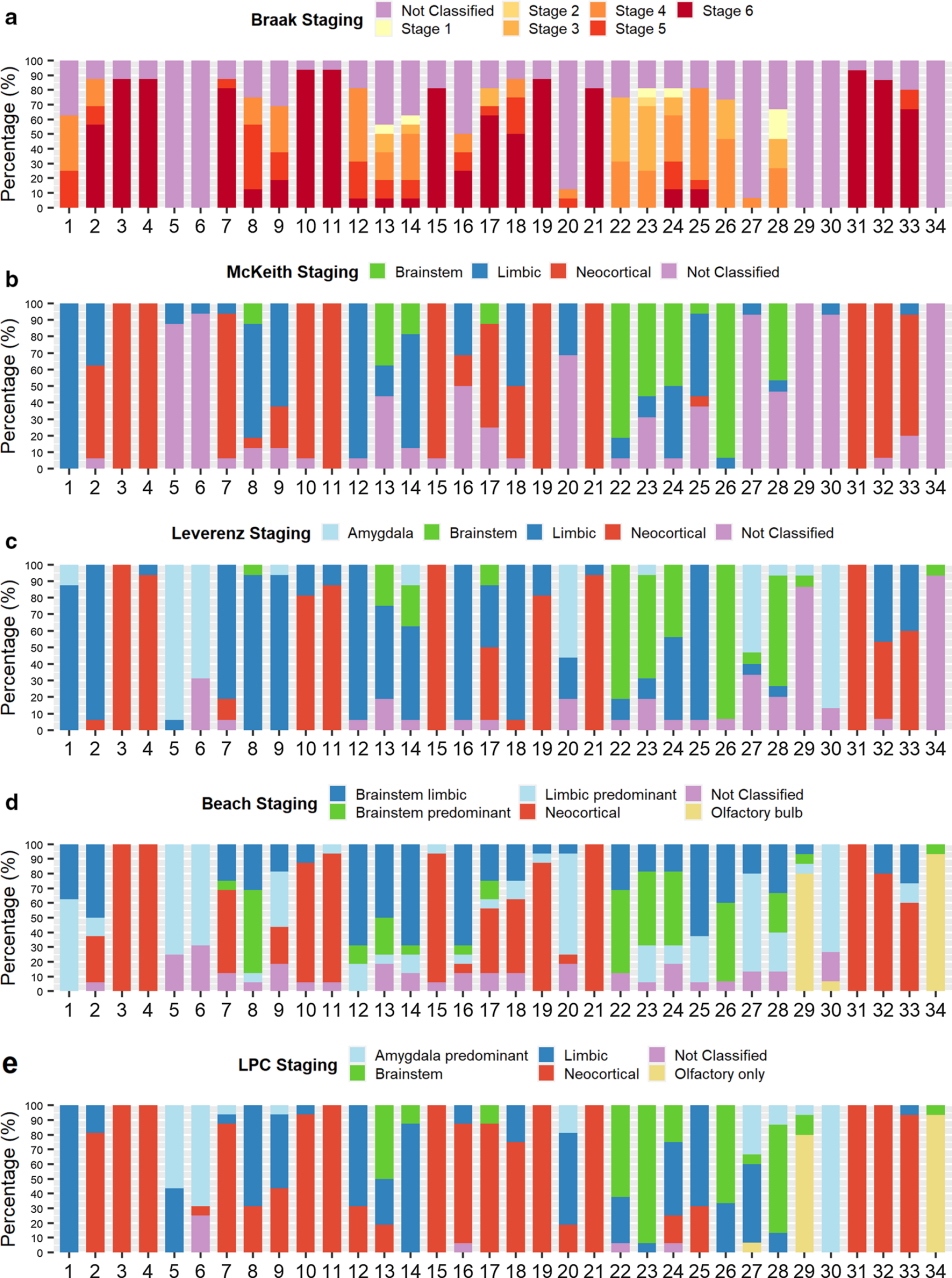
Percentages of assigned diagnostic categories according to Braak (**a**), McKeith (**b**), Leverenz (**c**), Beach (d) and LPC (**e**) systems. X-axis shows individual case numbers

Figure 4 presents the individual stages assigned to each case by the raters. Table 1 shows for each case the categories that reached highest agreement together with the percentage of raters who assigned this category as well as the mean agreement rates for each classification system. 100% agreement (including a ‘non-classifiable’ category) was reached in 14.7% of cases for Braak, 26.5% for McKeith, 8.8% for Leverenz, 11.8% for Beach, and 29.4% for LPC systems. Of note, when non-classifiable cases were excluded from the calculation, 100% agreement was never reached when using the Braak system (Table 1).

**Table 1:** Percentages of highest agreement in assigned categories

Case	Braak	McKeith	Leverenz	Beach	LPC	Clinical Diagnosis
Case 1	37.5% St. 4/ n.c	100% Limb	87.5% Lim	62.5% Lim.pr	100% Lim	Dementia FTD
Case 2	56.25% St. 6	56.25% Neoc	93.75% Lim	50% Brst.lim	81.25% Neoc	Dementia PDD
Case 3	87.5% St. 6	100% Neoc	100% Neoc	100% Neoc	100% Neoc	Dementia PDD
Case 4	87.5% St. 6	100% Neoc	93.75% Neoc	100% Neoc	100% Neoc	Dementia PDD
Case 5	100% n.c	87.5% n.c	93.75% Amy	75% Lim.pr	56.25% Amy.pr	Dementia AD
Case 6	100% n.c	93.75% n.c	68.75% Amy	68.75% Lim.pr	68.75% Amy.pr	Dementia AD
Case 7	81.25% St. 6	87.5% Neoc	81.25% Lim	56.25% Neoc	87.5% Neoc	Dementia PDD
Case 8	43.75% St. 5	68.75% Lim	93.75% Lim	56.25% Brst. pr	68.75% Lim	No Dem PD
Case 9	31.25% St. 4/ n.c	62.5% Lim	93.75% Lim	37.5% Lim.pr	50% Lim	No Dem MSA
Case 10	93.75% St.6	93.75% Neoc	81.25% Neoc	81.25% Neoc	93.75% Neoc	Dementia AD
Case 11	93.75% St. 6	100% Neoc	87.5% Neoc	87.5% Neoc	100% Neoc	Dementia DLB
Case 12	50% St. 4	93.75% Lim	93.75% Lim	68.75% Brst.lim	68.75% Lim	No Dem PD
Case 13	43.75% n.c	43.75% n.c	56.25% Lim	50% Brst.lim	50% Brst	Dementia AD
Case 14	37.5% n.c	68.75% Lim	56.25% Lim	68.75% Brst.lim	87.5% Lim	Dementia ADD
Case 15	81.25% St. 6	93.75% Neoc	100% Neoc.l	87.5% Neoc	100% Neoc	Dementia PDD
Case 16	50% n.c	50% n.c	93.75% Lim	68.75% Brst.lim	81.25% Neoc	Dementia ADD
Case 17	62.5% St. 6	62.5% Neoc	43.75% Neoc	43.75% Neoc	87.5% Neoc	Dementia PDD
Case 18	50% St. 6	50% Lim	93.75% Lim	50% Neoc	75% Neoc	Dementia DLB
Case 19	87.5% St. 6	100% Neoc	81.25% Neoc	87.5% Neoc	100% Neoc	Dementia PDD
Case 20	87.5% n.c	68.75% n.c	56.25% Amy	68.75% Lim.pr	62.5% Lim	Dementia ADD
Case 21	81.25% St. 6	100% Neoc	93.75% Neoc	100% Neoc	100% Neoc	Dementia PDD
Case 22	43.75% St. 3	81.25% Brst	81.25% Brst	56.25% Brst.pr	62.5% Brst	No Dem Control
Case 23	43.75% St. 3	56.25% Brst	62.5% Brst	0% Brst.pr	93.75% Brst	No Dem Control
Case 24	31.25% St. 4	50% Brst	50% Lim	50% Brst.pr	50% Lim	No Dem Control
Case 25	62.5% St. 4	50% Lim	93.75% Lim	62.5% Brst.lim	68.75% Lim	Dementia AD/DLB
Case 26	46.67% St. 4	93.33% Brst	93.33% Brst	53.33% Brst.pr	66.67% Brst	No Dem Control
Case 27	93.33% n.c	93.33% n.c	53.33% Amy	66.67% Lim.pr	53.33% Lim	Dementia AD
Case 28	33.33% n.c	46.67% Brst./ n.c	66.67% Brst	33.33% Brst.lim	73.33% Brst	No Dem Control
Case 29	100% n.c	100% n.c	86.67% n.c	80% Olf	80% Olf.only	Dementia AD
Case 30	100% n.c	93.33% n.c	86.67% Amy	73.33% Lim.pr	100% Amy.pr	Dementia AD
Case 31	93.33% St. 6	100% Neoc	100% Neoc	100% Neoc	100% Neoc	Dementia AD/DLB
Case 32	86.67% St. 6	93.33% Neoc	46.67% Lim./ Neoc	80% Neoc	100% Neoc	Dementia AD/DLB
Case 33	66.67% St. 6	73.33% Neoc	60% Neoc	60% Neoc	93.33% Neoc	Dementia DLB
Case 34	100% n.c	100% n.c	93.33% n.c	93.33% Olf	93.33% Olf.only	Dementia AD
Mean agreement	69%	79.80%	79.90%	68.50%	81%	
100% agreement including n.c	14.7%	26.5%	8.8%	11.8%	29.4%	
100% agreement excluding n.c	0%	29.2%%	9.4%	11.8%	29.4%	

*AD* Alzheimer’s disease; *Amy* amygdala; *Amy.pr* amygdala predominant; Beach, Unified Staging System for LBD by Beach and colleagues [3]; Braak, Lewy body Braak stages [5], Brst., brainstem; *Brst.lim* brainstem-limbic; *Brst.pr* brainstem predominant; *DLB* Dementia with Lewy bodies; *FTD* Frontotemporal dementia; *Leverenz* modified DLB consensus criteria by Leverenz and colleagues [10] *lim.* limbic; *lim.pr* limbic predominant; *LRPC* Lewy-related pathology consensus criteria; *McKeith* DLB Consensus Criteria by McKeith and colleagues [13]; *Neoc.* neocortical; *n.c,* non-classifiable; *Olf* olfactory; *PD* Parkinson’s disease; PDD, *PD* dementia

When Braak and McKeith categories were assigned using the dichotomized BrainNet Europe method, the inter-rater reliability increased for Braak (Krippendorff’s *α*: 0.47), while it remained virtually unchanged for McKeith (Krippendorff’s *α*: 0.57) systems. For both Braak and McKeith systems, the percentage of cases that were not classifiable decreased to 20.6% and 17.6% and 100% agreement rates increased considerably to 32.4% and 38.2%, respectively (Supplementary table 2, online resource).

### Evaluation of staging schemes in UPBB and NBTR archival cases

**Fig. 5:**
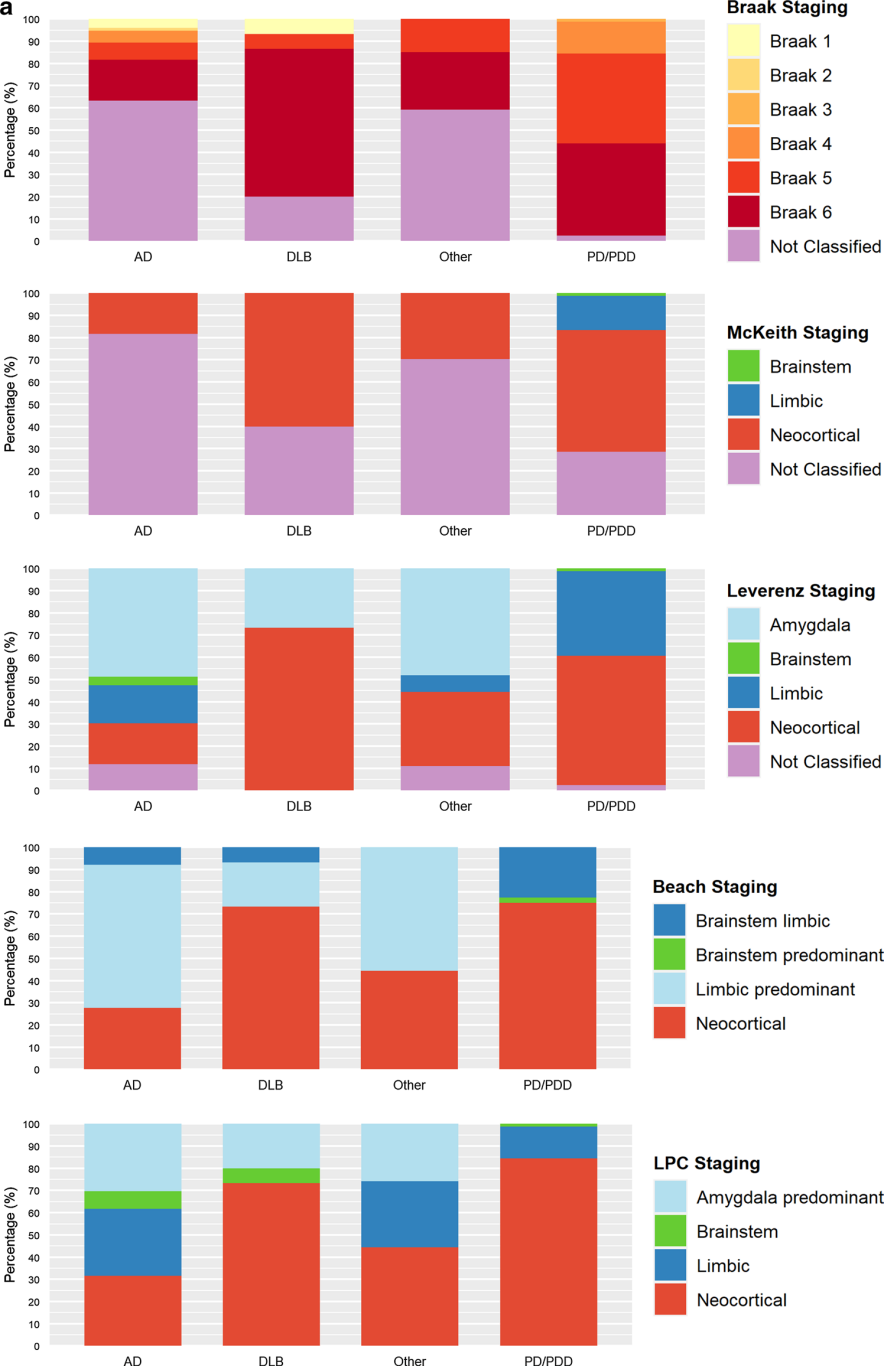

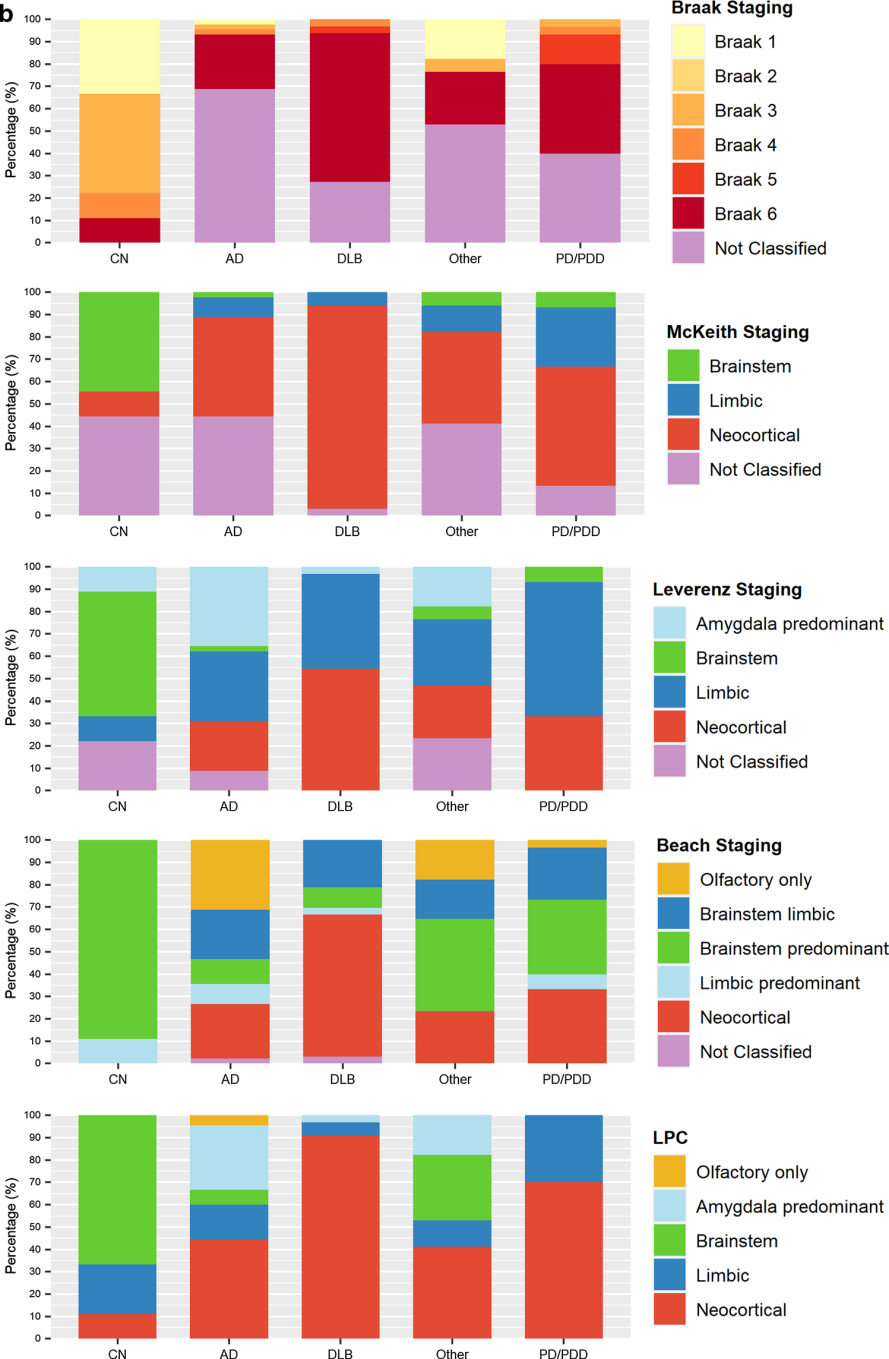
Diagnostic categories for archival cases of the University of Pennsylvania brain bank (UPBB; (**a**) and Newcastle Brain Tissue Resource (NBTR; **b**), stratified by their clinical diagnoses

We evaluated 202 UPBB and 134 NBTR archival cases. The most common clinic-pathological diagnoses were AD, DLB, PD and PDD. Figure 5 summarizes the neuropathological diagnoses, stratified by clinical diagnosis, assigned to the UPBB and NBTR cases. The Braak and McKeith systems yielded the largest number of cases that were non-classifiable, mainly for the AD dementia and “other diagnoses” group (59.3–69.9% for Braak and 41.2–81.6% for McKeith systems). The number of non-classifiable cases for the PD/PDD and DLB groups was lower for both systems (2.4–40% for Braak and 13.3%-40% for McKeith systems). Applying the Leverenz system led to a lower number of cases being non-classifiable (8.9–23.5% for the AD and “other diagnoses” and 0–8.9% for the DLB and PD/PDD). Most cases could be classified according to the Beach system (only 2.2% of AD and 3% of DLB NBTR cases were non-classifiable). All cases were classifiable by the LPC system. In the UPBB, two cases were considered to fit within two different Leverenz stages and one case to fit within two different McKeith stages. A comparison of the staging of cases in the different systems is summarized in Supplementary Tables 3 (UPBB) and 4 (NBTR).

Supplementary Tables 5 (UPBB; online resource) and 6 (NBTR; online resource) show the association between the different LPC categories and a dementia diagnosis. A possible limitation of the LPC is that the low neuropathological threshold needed to classify subjects as neocortical stage could lead to an “overcalling” of neocortical stages. To evaluate this possibility, we stratified cases by Braak neurofibrillary tangle stage and compared the odds of being demented at the time of death in patients with limbic versus neocortical LPC stages. Thus we evaluated if the neocortical stage was associated with greater odds of dementia compared to the limbic stage. The neocortical LPC stage was indeed associated with a 3.14 OR (*p* = 0.0001) of dementia in UPBB cases and a 5.0 OR (*p* < 0.0001) in NBTR cases. The difference between these ORs may be partly explained by differences in clinic-pathological diagnoses in the cohorts; in the UPBB cohort 9.5% and 28.6.% of cases had a clinic-pathological diagnosis of DLB and PD/PDD, respectively, while the NBTR cohort had a considerably higher percentage of DLB (37.8%) and completely lacked PD/PDD (Supplementary Table 1, online resource).

## Discussion

We have devised and tested a new staging system for the assessment of LP. Our proposed LPC system was applied together with previously established Braak, McKeith, Leverenz, and Beach systems, by 16 raters on 34 cases. The LPC system showed good inter-rater reliability: comparable to McKeith and Leverenz systems, and considerably better than Braak and Beach systems (Fig. 3b). Using the LPC system, the majority of raters were able to classify all cases; in comparison, while most cases (over 95%) could be classified using Beach, over 10% of cases could not be classified using Leverenz, over 25% using McKeith and nearly 30% using Braak systems, respectively (Fig. 3c). Percentages were even higher when UPBB and NBTR archival cases with a clinical diagnosis of AD dementia were evaluated (Fig. 5).

Since the initial identification of α-synuclein in LB [25], several staging systems have been proposed and implemented to classify LP [2, 3, 5, 14, 17]. The Braak system was developed to assess the typical patterns of severity and distribution of the LP in PD. However, later studies showed divergent patterns of progression in PD where the accumulation of pathological α-synuclein begins in the brainstem, as opposed to AD or DLB, where LP may be limited to limbic and neocortical regions [3, 30]. This helps explain the relatively high number of non-classifiable cases observed when applying the Braak system in our study. The McKeith system showed a similar high percentage of non-classifiable cases, partly reflecting the necessity to have at least some brainstem pathology to assign any stage, which is also true for the Leverenz system. In addition, according to the McKeith system, some cases can equally fulfil the criteria for limbic and neocortical LP (e.g., brainstem and limbic regions, score 3; temporal cortex score 2 and frontal cortex score 1); consequently, such cases cannot be assigned to just a single category and thus are not classifiable. Both Braak [5] and McKeith [17] systems were published before it was shown that LP may be restricted to the olfactory bulb or amygdala [2, 3, 13] and, therefore, such cases cannot be assigned a category in both Braak and McKeith systems. However, in our study, only three cases were categorized as “Amygdala predominant” and one as “Olfactory only”. While application of the method suggested by the BrainNet Europe [2] resulted in a reduction of percentage rates of cases that could not be classified, they were still higher than for all other systems.

Assignment of a category in both Braak and Beach systems depends heavily on the semi-quantitative score for LP in each region. Since that is relatively subjective, it is not surprising that both Braak and Beach systems had the lowest inter-rater reliability in our study (Fig. 3c). Semi-quantitative scores are also used in McKeith and Leverenz systems, but regional scores may range from 1 to 3 and individual scores do not, therefore, influence the assignment of a category as much as they do in Braak and Beach systems. We have seen a high inter-rater reliability for both McKeith and Leverenz systems as well as for our proposed LPC system; the use of a dichotomized approach where a region can either be scored negative or positive for LP greatly reduces the probability of differences in scores between multiple raters. This is further supported by our finding of Braak systems showing higher inter-rater reliability and both Braak and McKeith system showing highest percentage of cases with 100% agreement, when the dichotomized method suggested by the BrainNet Europe was used. However, 100% agreement was only reached in 29.4% when using the LPC system, which is still higher than the 100% agreement rates for Braak, McKeith, Leverenz, and Beach systems, but admittedly relatively low considering the dichotomized scoring and the simple staging approach. We assume that the use of only digital images had an adverse impact on the scoring accuracy of raters, who are used to assessing slides on a microscope, in particular since sometimes relatively large areas had to be screened for minimal amounts of pathology (e.g., single LNs in a neocortical section).

In addition to our multi-rater assessment, we evaluated the LPC system in comparison with Braak, McKeith, Leverenz, and Beach systems, in a total of 336 archival cases from the UPBB and NBTR: a large sample of consecutive non-selected cases with a broad range of clinical diagnoses. LP in PD cases with or without cognitive impairment was classifiable by all staging systems. However, when dementia was the main presenting feature, LP was not classifiable in 41–82% of cases staged according to Braak or McKeith systems (Fig. 5). This inability to stage a high proportion of cases according to Braak or McKeith systems is in keeping with previous findings by Beach and colleagues [3]. Both Beach and our proposed LPC system are better suited for the classification of LP pathology across the entire spectrum of neurodegenerative diseases and ageing.

We scored a region positive if sparse LBs or LNs were seen thereby giving equal importance to LBs and LNs for assigning the lowest possible positive LP score, which is in agreement with previous publications on the assessment of LP in post-mortem brains [2, 3, 14, 17]. Hence, our dichotomous LP scoring approach leads to cases with relatively low amounts of LP in limbic/neocortical areas being categorised as limbic/neocortical LP. While this could in theory possibly result in a relatively high proportion of cognitively unimpaired individuals being diagnosed as having neocortical LP, in the multi-rater assessment all 15 cases with neocortical LP, as determined by the majority of raters, had a clinical diagnosis of dementia. Moreover, in both UPBB and NBTR, a LPC category of neocortical LP was associated with significantly increased odds of having dementia in life even after controlling for neurofibrillary tangle tau pathology. However, some α-synuclein antibodies may produce non-specific immunolabelling [8] and, therefore, we suggest that the presence of single dot-like immunopositivity in the neuropil alone in the absence of any neuronal immunopositivity is not sufficient to score the section positive (Fig. 2a, b). We further suggest that detailed clinico-pathological correlative studies should not be based on diagnostic staging systems, like the one we present here, but always aim to obtain more quantitative measures of the burden of pathological protein aggregates (*e.g.*, image analysis).

To make our system applicable for neuropathological routine diagnostics at relatively low costs, we have deliberately limited the number of regions that need to be assessed to an absolute minimum and have chosen those regions that have been widely used in previous staging systems. However, LP in particular in PD, may be present in a variety of tissues such as the spinal cord [7], gut [6, 27], sympathetic ganglia [26], adrenal gland [11], heart [22], and skin [9] among others. The systematic pathological assessment of LP in regions outside the brain may be possible in the future if post-mortem examination related to neurodegeneration routinely combines assessment of both cerebral and relevant extra-cerebral tissues, and will lead to the development of staging systems for LP that encompass LP in the entire human body.

In our study, two different antibodies were used, the KM51 clone (Leica, UK), which detects full length α-synuclein was used for NBTR cases while UPBB cases were stained with Syn303 (CNDR) which detects epitopes with amino acid residues 2–4. We did not observe any differences in inter-rater reliability or ability to classify cases between cases from NBTR and UPBB, suggesting that the reliability of LPC is not dependent on specific α-synuclein antibodies.

The LPC system was devised primarily to increase the reliability of diagnostic assessment, without implying any particular pattern of topographical spread of pathology, such as in the Beach system [1, 3]. Our findings confirm that the Beach system, based on the putative pathological processes underlying disease progression, allows most cases to be staged and is, therefore, a useful scheme if used by experienced raters, although due to the low inter-rater reliability it may not practicable for day-to-day routine diagnostics and collection of data across brain bank networks. We would also note that we did not include the assessment of substantia nigra cell loss in the inter-rater evaluation as this is not included in previous LP staging systems and was not within the aims of our study. However, we suggest that evaluation of substantia nigra cell loss should routinely be performed, as previously recommended by the BrainNet Europe Consortium [2]. The Fourth Consensus Report of the DLB Consortium further suggests to score nigral neuronal cell loss to subclassify cases into those likely or not to have Parkinsonism and the LPC categories can be used to determine the likelihood that pathological findings are associated with a typical DLB clinical syndrome (Table 2 in [18]).

We used the term LP instead of LBD in the LPC system categories and we recommend that the terms PD-MCI, PDD or DLB not be used to describe the neuropathological findings alone. These diagnoses should only be made once the clinical presentation, including neuropsychological evaluation, is combined with the post-mortem neuropathological findings. In addition, as the ageing brain typically includes multiple pathologies which together can lower the threshold for one specific pathology to cause dementia (or other neurological impairment) [4, 12, 28], the neuropathological report should contain information on all observed pathologies, *e.g.,* AD neuropathological change [19], TDP-43 pathology [16, 21], cerebrovascular pathology [24], and LP.

We conclude that the LPC system is a useful classification system for LP. It has good reproducibility and clinical utility, and our expectation is that it will be reliable and useful in routine diagnostic practice, allowing neuropathologists to classify the majority of cases into categories that are compatible with the clinical findings. We suggest that the LPC system should be the standard future approach for the basic post-mortem evaluation of LP in individuals with and without concomitant neurodegenerative diseases.

## Supplementary Information

Below is the link to the electronic supplementary material.

Supplementary file1 (DOCX 6898 KB)

## References

[1] Adler CH, Beach TG, Zhang N (2019). Unified staging system for lewy body disorders: clinicopathologic correlations and comparison to braak staging. J Neuropathol Exp Neurol.

[2] Alafuzoff I, Ince PG, Arzberger T (2009). Staging/typing of lewy body related alpha-synuclein pathology: a study of the BrainNet Europe Consortium. Acta Neuropathol.

[3] Beach TG, Adler CH, Lue L (2009). Unified staging system for Lewy body disorders: correlation with nigrostriatal degeneration, cognitive impairment and motor dysfunction. Acta Neuropathol.

[4] Boyle PA, Yu L, Leurgans SE (2019). Attributable risk of Alzheimer’s dementia attributed to age-related neuropathologies. Ann Neurol.

[5] Braak H, Del Tredici K, Rub U, de Vos RA, Jansen Steur EN, Braak E (2003). Staging of brain pathology related to sporadic Parkinson’s disease. Neurobiol Aging.

[6] Braak H, de Vos RA, Bohl J, Del Tredici K (2006). Gastric alpha-synuclein immunoreactive inclusions in Meissner’s and Auerbach’s plexuses in cases staged for Parkinson's disease-related brain pathology. Neurosci Lett.

[7] Braak H, Sastre M, Bohl JR, de Vos RA, Del Tredici K (2007). Parkinson’s disease: lesions in dorsal horn layer I, involvement of parasympathetic and sympathetic pre- and postganglionic neurons. Acta Neuropathol.

[8] Delic V, Chandra S, Abdelmotilib H (2018). Sensitivity and specificity of phospho-Ser129 alpha-synuclein monoclonal antibodies. J Comp Neurol.

[9] Doppler K, Ebert S, Uceyler N (2014). Cutaneous neuropathy in Parkinson’s disease: a window into brain pathology. Acta Neuropathol.

[10] Emre M, Aarsland D, Brown R (2007). Clinical diagnostic criteria for dementia associated with Parkinson’s disease. Mov Disord.

[11] Fumimura Y, Ikemura M, Saito Y (2007). Analysis of the adrenal gland is useful for evaluating pathology of the peripheral autonomic nervous system in lewy body disease. J Neuropathol Exp Neurol.

[12] Irwin DJ, White MT, Toledo JB (2012). Neuropathologic substrates of Parkinson disease dementia. Ann Neurol.

[13] Jellinger KA (2004). Lewy body-related alpha-synucleinopathy in the aged human brain. J Neural Transm.

[14] Leverenz JB, Hamilton R, Tsuang DW (2008). Empiric refinement of the pathologic assessment of Lewy-related pathology in the dementia patient. Brain Pathol.

[15] Litvan I, Goldman JG, Troster AI (2012). Diagnostic criteria for mild cognitive impairment in Parkinson’s disease: movement disorder society task force guidelines. Mov Disord.

[16] Mackenzie IR, Neumann M, Baborie A (2011). A harmonized classification system for FTLD-TDP pathology. Acta Neuropathol.

[17] McKeith IG, Dickson DW, Lowe J (2005). Diagnosis and management of dementia with Lewy bodies: third report of the DLB Consortium. Neurology.

[18] McKeith IG, Boeve BF, Dickson DW (2017). Diagnosis and management of dementia with Lewy bodies: Fourth consensus report of the DLB Consortium. Neurology.

[19] Montine TJ, Phelps CH, Beach TG (2012). National Institute on aging-alzheimer’s association guidelines for the neuropathologic assessment of alzheimer’s disease: a practical approach. Acta Neuropathol.

[20] Muller CM, de Vos RA, Maurage CA, Thal DR, Tolnay M, Braak H (2005). Staging of sporadic Parkinson disease-related alpha-synuclein pathology: inter- and intra-rater reliability. J Neuropathol Exp Neurol.

[21] Nelson PT, Dickson DW, Trojanowski JQ (2019). Limbic-predominant age-related TDP-43 encephalopathy (LATE): consensus working group report. Brain.

[22] Orimo S, Ghebremedhin E, Gelpi E (2018). Peripheral and central autonomic nervous system: does the sympathetic or parasympathetic nervous system bear the brunt of the pathology during the course of sporadic PD?. Cell Tissue Res.

[23] Postuma RB, Berg D, Stern M (2015). MDS clinical diagnostic criteria for Parkinson’s disease. Mov Disord.

[24] Skrobot OA, Attems J, Esiri M (2016). Vascular cognitive impairment neuropathology guidelines (VCING): the contribution of cerebrovascular pathology to cognitive impairment. Brain.

[25] Spillantini MG, Schmidt ML, Lee VM, Trojanowski JQ, Jakes R, Goedert M (1997). Alpha-synuclein in Lewy bodies. Nature.

[26] Takeda S, Yamazaki K, Miyakawa T, Arai H (1993). Parkinson's disease with involvement of the parasympathetic ganglia. Acta Neuropathol.

[27] Tanei ZI, Saito Y, Ito S (2020). Lewy pathology of the esophagus correlates with the progression of Lewy body disease: a Japanese cohort study of autopsy cases. Acta Neuropathol.

[28] Toledo JB, Arnold SE, Raible K (2013). Contribution of cerebrovascular disease in autopsy confirmed neurodegenerative disease cases in the National Alzheimer's Coordinating Centre. Brain.

[29] Toledo JB, Van Deerlin VM, Lee EB (2014). A platform for discovery: The University of Pennsylvania integrated neurodegenerative disease Biobank. Alzheimers Dement.

[30] Toledo JB, Gopal P, Raible K (2016). Pathological alpha-synuclein distribution in subjects with coincident Alzheimer’s and Lewy body pathology. Acta Neuropathol.

