# Supplementary material for: Neuropathological consensus criteria for the evaluation of Lewy pathology in post-mortem brains: a multi-centre study
Source: Acta Neuropathol. 2021 Jan 5;141(2):159–72. doi: 10.1007/s00401-020-02255-2 (PMC7847437; doi:10.1007/s00401-020-02255-2)

**Supplementary table 1**. Autopsy cases from the University of Pennsylvania Brain Bank (UPBB) and Newcastle Brain Tissue Resource (NBTR) brain banks included in the multi-rater assessment of the staging criteria.

|  | | UPBB | NBTR |
| --- | --- | --- | --- |
| Age at death | | 79 [74-85] | 86 [70-102] |
| Gender (% male) | | 71.40% | 53.84% |
| Clinico-pahological  diagnosis | AD | 28.60% | 30.77% |
|  | DLB | 9.50% | 30.77% (incl. Mix AD/DLB) |
|  | Other | 33.30% | 38.46% (all controls) |
|  | PD/PDD | 28.60% | 0% |
| CERAD score | neg | 19% | 30.77% |
|  | A | 0% | 15.38% |
|  | B | 23.80% | 0% |
|  | C | 57.10% | 53.85% |
| NFT Braak stage | 0 | 0% | 0% |
|  | I-II | 27.80% | 46.15% |
|  | III-IV | 16.70% | 0% |
|  | V-VI | 55.60% | 53.85% |

Abbreviations: AD, Alzheimer's disease; NFT Braak stage, Braak staging of neurofibrillary tangles; CERAD, Consortium to establish a registry for Alzheimer's disease; DLB, dementia with Lewy bodies; PD, Parkinson's disease; PDD, Parkinson's disease dementia.

**Supplementary Table 2**. Percentages of highest agreement in assigned categories applying the dichotomized scoring method of BrainNet Europe to Braak and Mckeith systems (Alafuzoff I et al (2009) Acta Neuropathol 117:635-652).

| **Case** | **Braak** | **McKeith** |
| --- | --- | --- |
| Case 1 | 92.31% Stage 5 | 100% Limbic |
| Case 2 | 84.62% Stage 6 | 84.62% Neocortical |
| Case 3 | 100% Stage 6 | 100% Neocortical |
| Case 4 | 100% Stage 6 | 100% Neocortical |
| Case 5 | 92.31% Not classified | 92.31% Not classified |
| Case 6 | 100% Not classified | 100% Not classified |
| Case 7 | 100% Stage 6 | 100% Neocortical |
| Case 8 | 53.85% Stage 5 | 53.85% Limbic |
| Case 9 | 46.15% Stage 6 | 46.15% Limbic |
| Case 10 | 100% Stage 6 | 100% Neocortical |
| Case 11 | 100% Stage 6 | 100% Neocortical |
| Case 12 | 38.46% Stage 5/ Stage 6 | 61.54% Limbic |
| Case 13 | 30.77% Stage 5 | 61.54% Limbic |
| Case 14 | 84.62% Stage 5 | 92.31% Limbic |
| Case 15 | 100% Stage 6 | 100% Neocortical |
| Case 16 | 76.92% Stage 6 | 84.62% Neocortical |
| Case 17 | 76.92% Stage 6 | 83.33% Neocortical |
| Case 18 | 76.92% Stage 6 | 76.92% Neocortical |
| Case 19 | 92.31% Stage 6 | 100% Neocortical |
| Case 20 | 92.31% Not classified | 53.85% Not classified |
| Case 21 | 92.31% Stage 6 | 100% Neocortical |
| Case 22 | 53.85% Stage 3 | 53.85% Brainstem |
| Case 23 | 69.23% Stage 3 | 84.62% Brainstem |
| Case 24 | 38.46% Stage 5 | 53.85% Limbic |
| Case 25 | 53.85% Stage 5 | 69.23% Limbic |
| Case 26 | 38.46% Stage 3/ Stage 4 | 69.23% Limbic |
| Case 27 | 84.62% Not classified | 69.23% Not classified |
| Case 28 | 46.15% Stage 3 | 76.92% Brainstem |
| Case 29 | 84.62% Not classified | 84.62% Not classified |
| Case 30 | 100% Not classified | 92.31% Not classified |
| Case 31 | 100% Stage 6 | 100% Neocortical |
| Case 32 | 100% Stage 6 | 100% Neocortical |
| Case 33 | 92.31% Stage 6 | 92.31% Neocortical |
| Case 34 | 100% Not classified | 100% Not classified |
| Mean agreement (%) | 79.20% | 83.40% |
| Cases with 100% agreement | 32.40% | 38.20% |
| Cases with 100% agreement excluding non classifiable cases | 29.60% | 40.70% |

**Supplementary table 3.** Comparing previous staging systems with the Lewy pathology consensus criteria (LPC) for the University of Pennsylvania Brain Bank.

|  |  | LPC | | | |
| --- | --- | --- | --- | --- | --- |
| System | Stage | Amygdala predominant | Brainstem | Limbic | Neocortical |
| Braak | n.c. | 33 | 2 | 26 | 8 |
|  | 1 | 0 | 4 | 0 | 0 |
|  | 2 | 0 | 1 | 0 | 0 |
|  | 3 | 0 | 1 | 0 | 0 |
|  | 4 | 0 | 0 | 13 | 3 |
|  | 5 | 0 | 0 | 3 | 42 |
|  | 6 | 0 | 0 | 1 | 65 |
| McKeith | n.c. | 33 | 7 | 35 | 36 |
|  | Brainstem | 0 | 1 | 0 | 0 |
|  | Limbic | 0 | 0 | 8 | 5 |
|  | Neocortical | 0 | 0 | 0 | 77 |
| Leverenz | n.c | 0 | 2 | 5 | 7 |
|  | Amygdala predominant | 33 | 2 | 19 | 0 |
|  | Brainstem | 0 | 4 | 0 | 0 |
|  | Limbic | 0 | 0 | 19 | 28 |
|  | Neocortical | 0 | 0 | 0 | 83 |
| Beach | n.c. | 0 | 0 | 0 | 0 |
|  | Brainstem  predominant | 0 | 1 | 1 | 0 |
|  | Brainstem-  Limbic | 0 | 5 | 10 | 11 |
|  | Limbic predominant | 33 | 2 | 28 | 4 |
|  | Neocortical | 0 | 0 | 4 | 103 |

**Supplementary table 4.** Comparing previous staging systems with the Lewy pathology consensus criteria (LPC) for the Newcastle Brain Tissue Resource.

|  |  | LPC | | | | |
| --- | --- | --- | --- | --- | --- | --- |
| System | Stage | Amygdala predominant | Olfactory only | Brainstem | Limbic | Neocortical |
| Braak | n.c. | 17 | 2 | 2 | 11 | 29 |
|  | 1 | 0 | 0 | 7 | 0 | 0 |
|  | 3 | 0 | 0 | 5 | 2 | 0 |
|  | 4 | 0 | 0 | 0 | 4 | 0 |
|  | 5 | 0 | 0 | 0 | 5 | 0 |
|  | 6 | 0 | 0 | 0 | 0 | 50 |
| McKeith | n.c. | 17 | 2 | 9 | 4 | 4 |
|  | Brainstem | 0 | 0 | 5 | 3 | 0 |
|  | Limbic | 0 | 0 | 0 | 15 | 1 |
|  | Neocortical | 0 | 0 | 0 | 0 | 74 |
| Leverenz | n.c | 0 | 2 | 6 | 2 | 0 |
|  | Amygdala predominant | 17 | 0 | 3 | 1 | 0 |
|  | Brainstem | 0 | 0 | 5 | 4 | 0 |
|  | Limbic | 0 | 0 | 0 | 15 | 37 |
|  | Neocortical | 0 | 0 | 0 | 0 | 42 |
| Beach | n.c. | 2 | 0 | 0 | 0 | 0 |
|  | Olfactory only | 15 | 2 | 0 | 0 | 1 |
|  | Brainstem  predominant | 0 | 0 | 14 | 11 | 8 |
|  | Brainstem-  Limbic | 0 | 0 | 0 | 5 | 22 |
|  | Limbic predominant | 0 | 0 | 0 | 6 | 2 |
|  | Neocortical | 0 | 0 | 0 | 0 | 46 |

n.c.= non-classifiable

**Supplementary table 5.** Presence of dementia based on Braak neurofibrillary tangle (NFT) stage and Lewy pathology consensus criteria (LPC) category for the University of Pennnnsylvania Brain Bank. Percentages of cases without and with dementia diagnosis based on NFT Braak and LRPC staging systems.

| Braak NFT |  | Dementia | | Sum |
| --- | --- | --- | --- | --- |
|  | LPC | No | Yes |  |
| 0 | Amygdala predominant | 0% | 0% | 0% |
|  | Brainstem | 0% | 0% | 0% |
|  | Limbic | 37.5% | 12.5% | 50% |
|  | Neocortical | 0% | 50% | 50% |
|  | Sum | 37.5% | 62.5% | 100% |
| I-II | Amygdala predominant | 0% | 0% | 0% |
|  | Brainstem | 0% | 0% | 0% |
|  | Limbic | 11.63% | 2.33% | 13.95% |
|  | Neocortical | 27.91% | 58.14% | 86.05% |
|  | Sum | 39.53% | 60.47% | 100% |
| III-IV | Amygdala predominant | 0% | 3.23% | 3.23% |
|  | Brainstem | 0% | 6.45% | 6.45% |
|  | Limbic | 0% | 3.23% | 3.23% |
|  | Neocortical | 9.68% | 77.42% | 87.1% |
|  | Sum | 9.68% | 90.32% | 100% |
| V-VI | Amygdala predominant | 0% | 27.12% | 27.12% |
|  | Brainstem | 0% | 5.08% | 5.08% |
|  | Limbic | 0.85% | 26.27% | 27.12% |
|  | Neocortical | 1.69% | 38.98% | 40.68% |
|  | Sum | 2.54% | 97.46% | 100% |

**Supplementary table 6.** Presence of dementia based on Braak neurofibrillary tangle (NFT) stage and Lewy pathology consensus criteria (LPC) category for the NBTR brain bank. Percentages of cases without and with dementia diagnosis based on NFT Braak and LPC staging systems.

| Braak NFT |  | Dementia | | Sum |
| --- | --- | --- | --- | --- |
|  | LPC | No | Yes |  |
| 0 | Amygdala predominant | 0% | 0% | 0% |
|  | Olfactory only | 0% | 0% | 0% |
|  | Brainstem | 33.33% | 0% | 33.33% |
|  | Limbic | 0% | 0% | 0% |
|  | Neocortical | 33.33% | 33.33% | 66.67% |
|  | Sum | 66.67% | 33.33% | 100% |
| I-II | Amygdala predominant | 0% | 3.33% | 3.33% |
|  | Olfactory only | 0% | 0% | 0% |
|  | Brainstem | 16.67% | 0% | 16.67% |
|  | Limbic | 23.33% | 3.33% | 26.67% |
|  | Neocortical | 10% | 43.33% | 53.33% |
|  | Sum | 50% | 50% | 100% |
| III-IV | Amygdala predominant | 0% | 2.56% | 2.56% |
|  | Olfactory only | 0% | 0% | 0% |
|  | Brainstem | 7.69% | 2.56% | 10.26% |
|  | Limbic | 10.26% | 2.56% | 12.82% |
|  | Neocortical | 7.69% | 66.67% | 74.36% |
|  | Sum | 25.64% | 74.36% | 100% |
| V-VI | Amygdala predominant | 0% | 24.59% | 24.59% |
|  | Olfactory only | 0% | 3.28% | 3.28% |
|  | Brainstem | 1.64% | 4.92% | 6.56% |
|  | Limbic | 0% | 14.75% | 14.75% |
|  | Neocortical | 0% | 50.82% | 50.82% |
|  | Sum | 1.64% | 98.36% | 100% |

**Supplementary figure 1.** Olfactory bulb section scores**.**


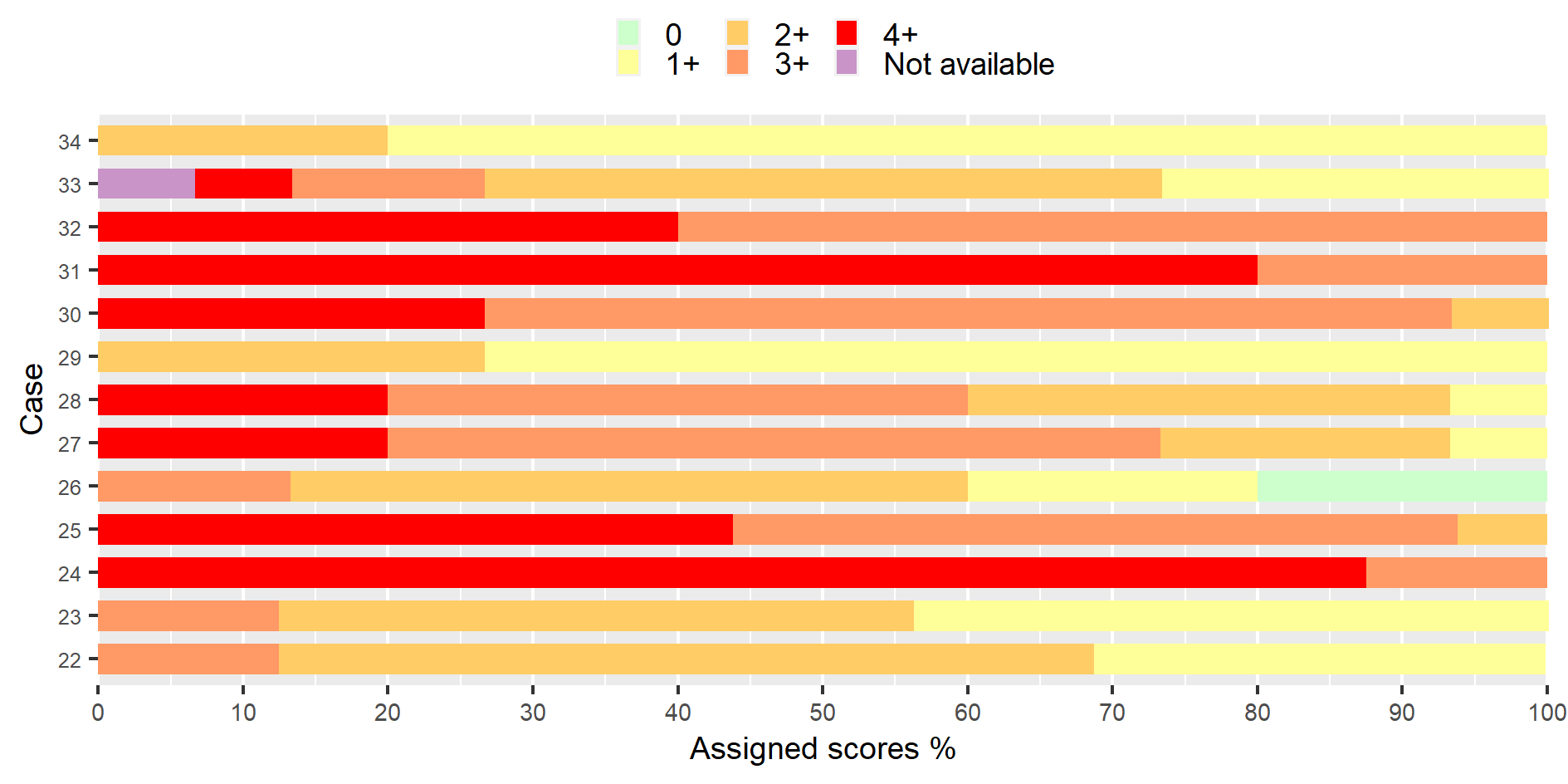


**Supplementary figure 2.** Medulla section scores. For case 21 a medulla section was not available.


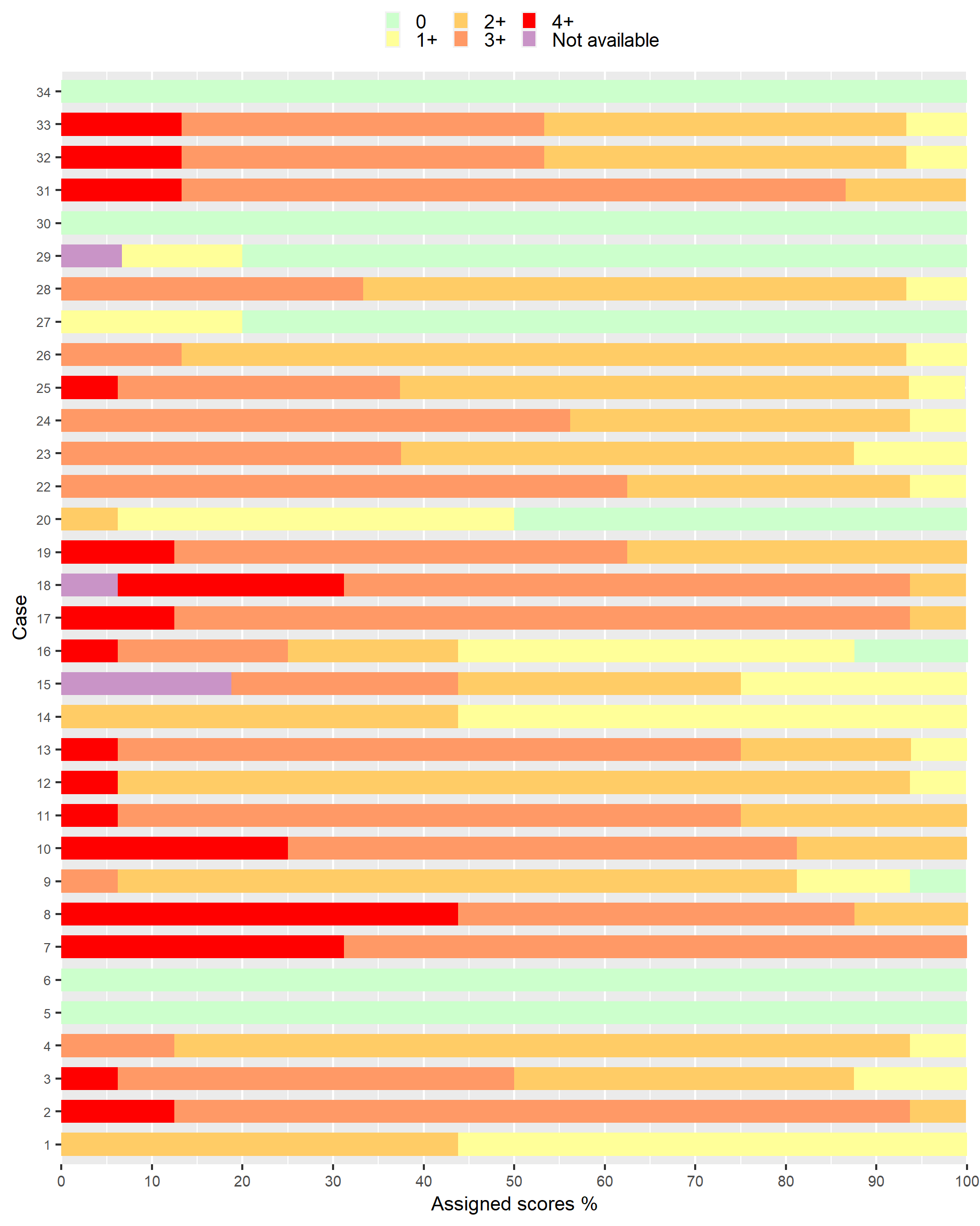


**Supplementary figure 3.** Substantia nigra section scores.


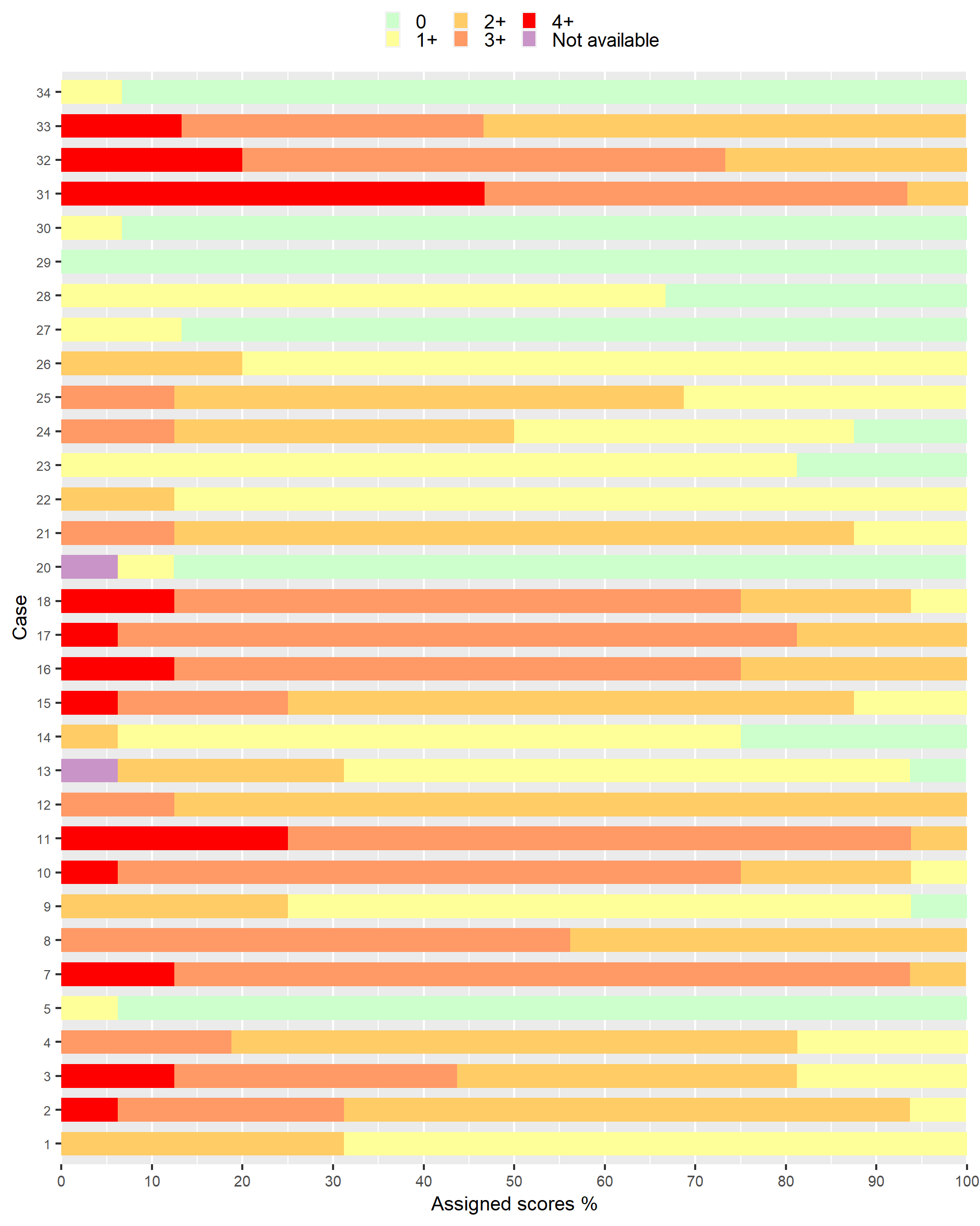


**Supplementary figure 4.** Amygdala section scores.


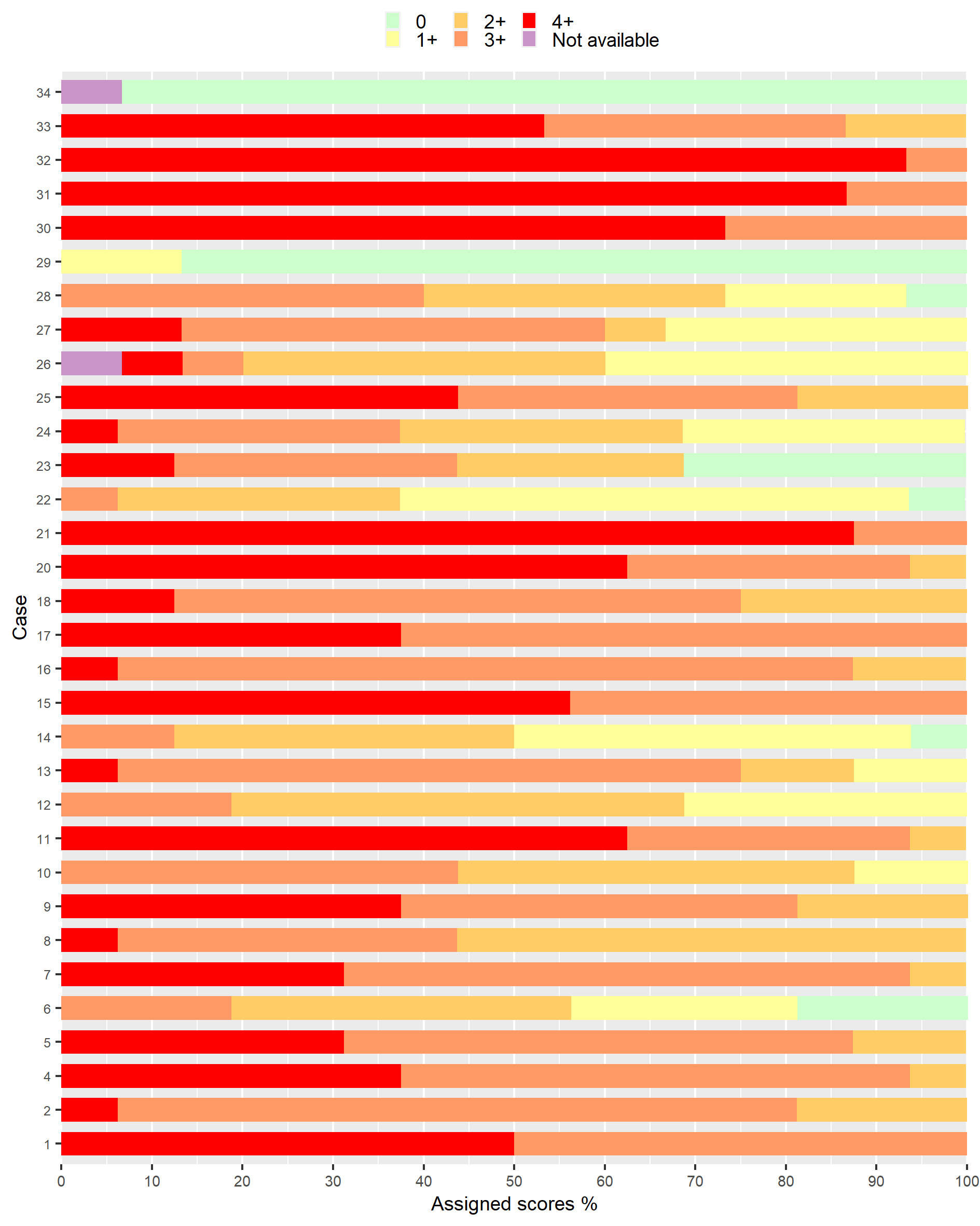


**Supplementary figure 5.** Cingulate cortex scores.

**
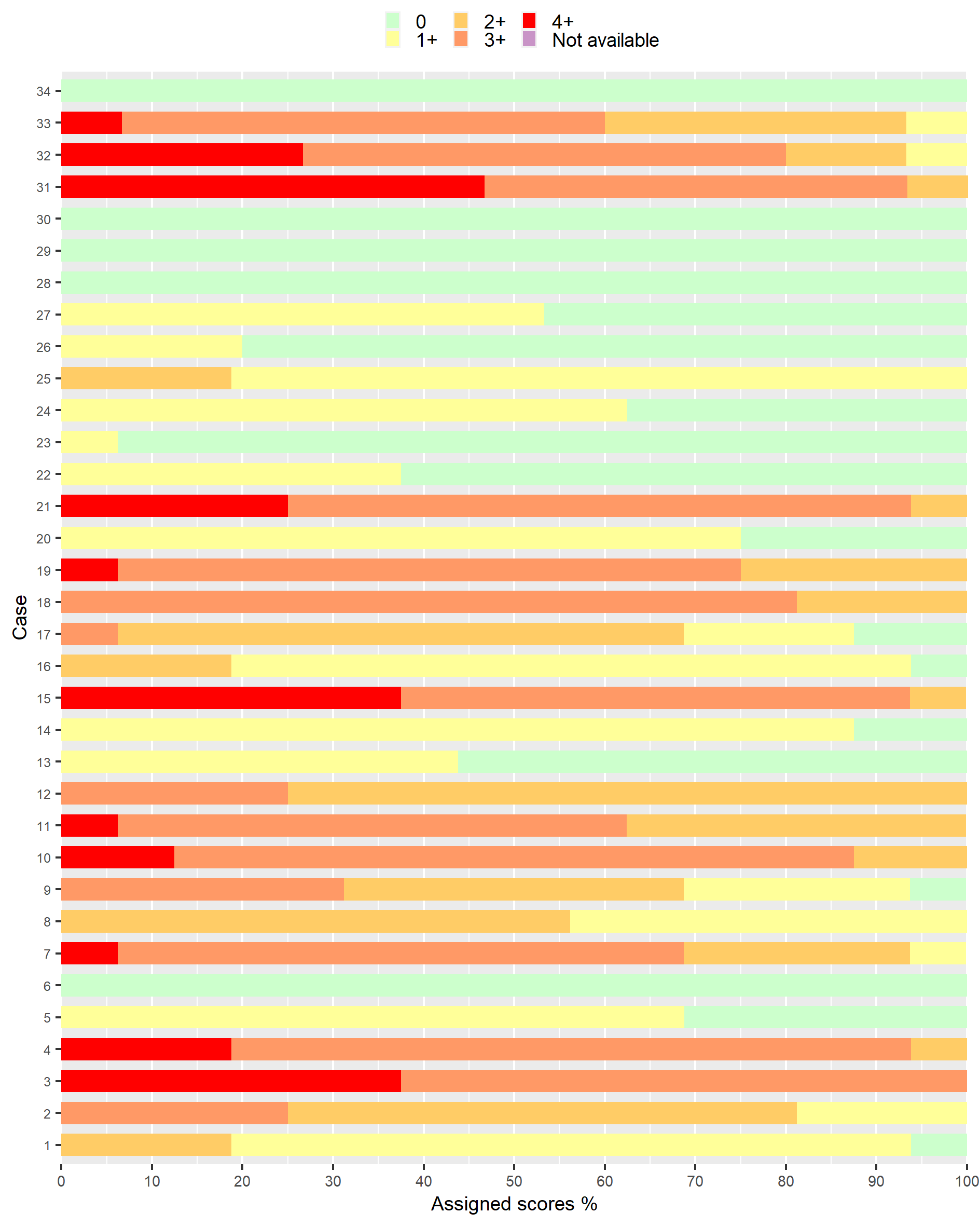
**

**Supplementary figure 6.** Parahippocampal gyrus section scores.


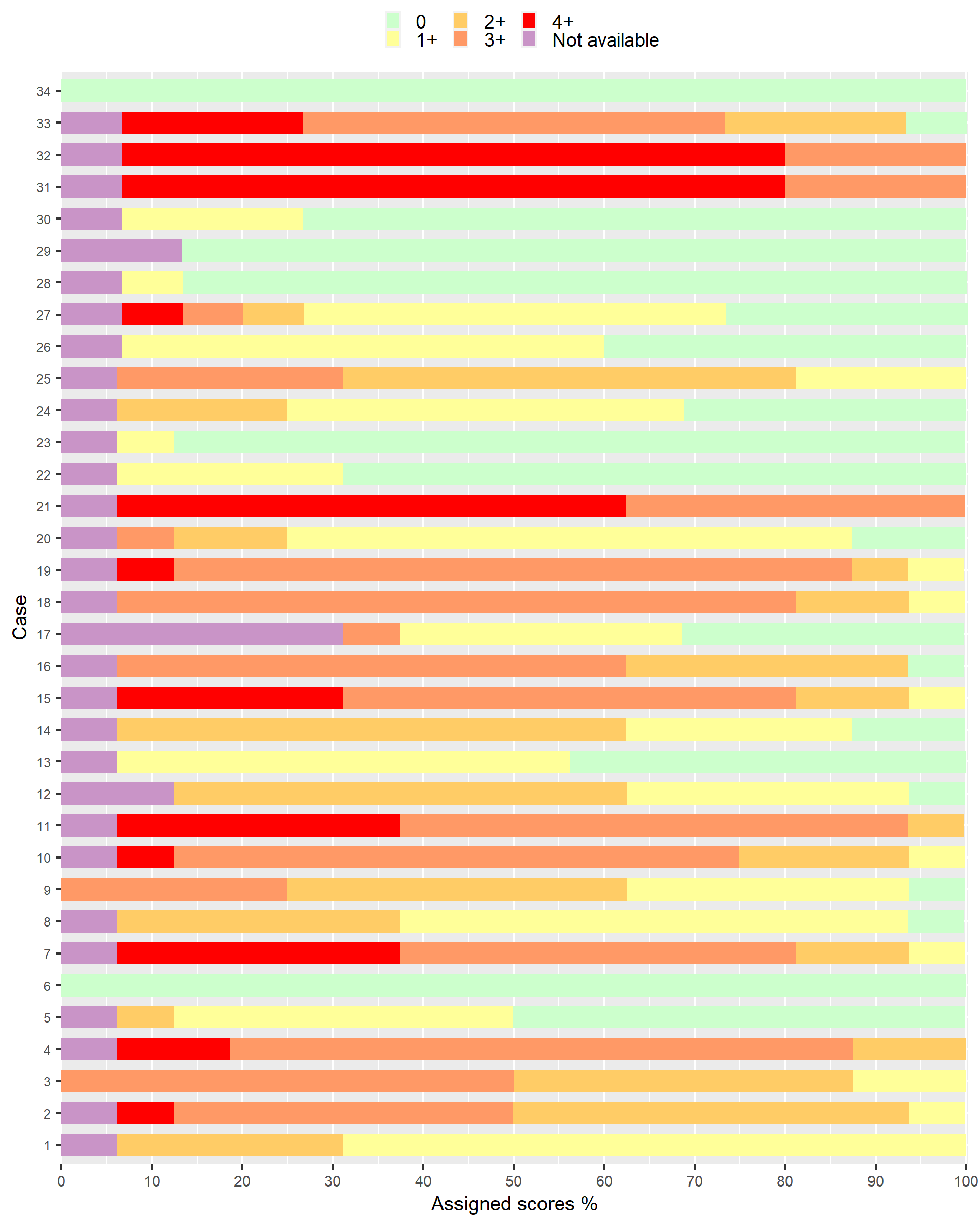


**Supplementary figure 7.** Temporal cortex section scores.


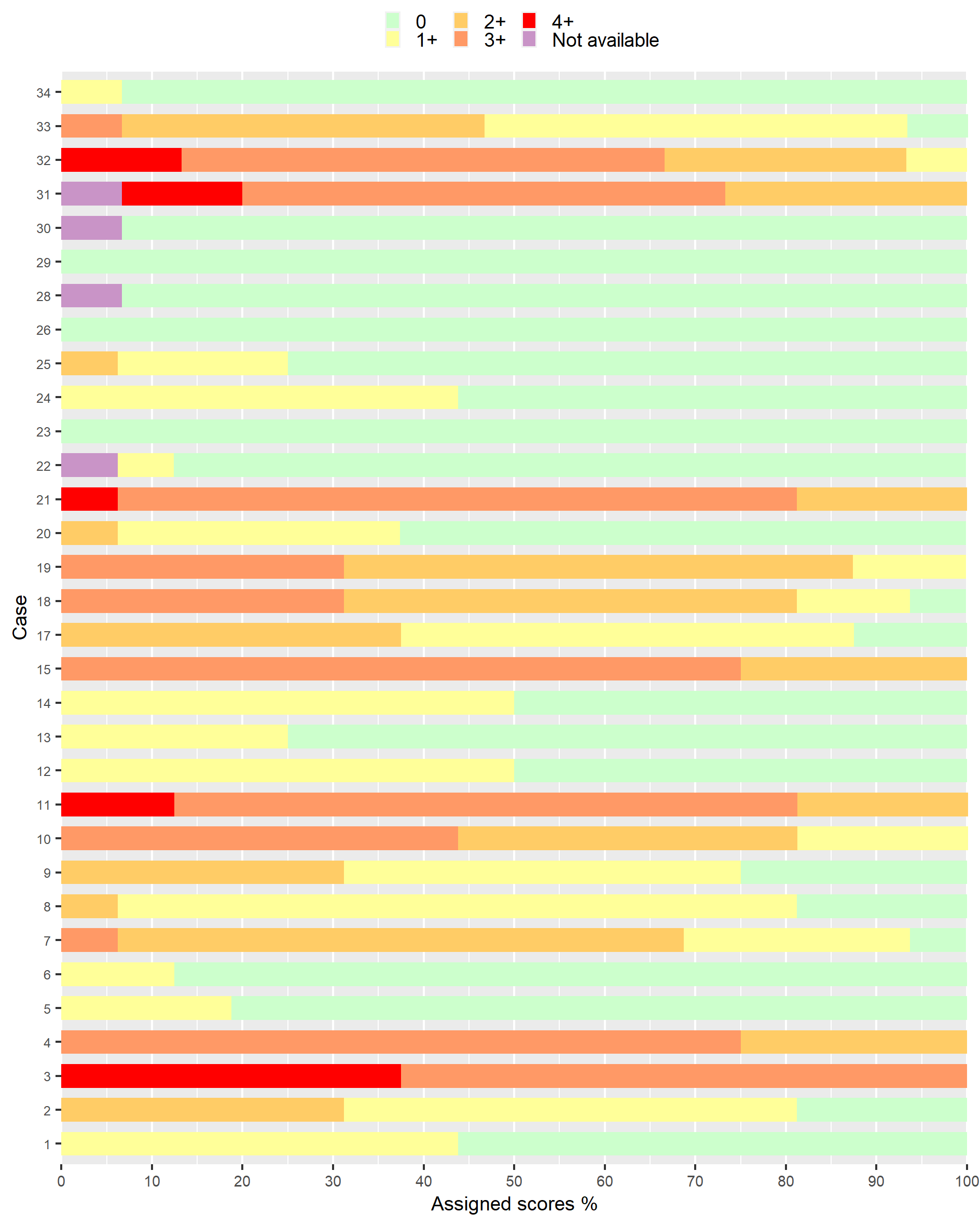


**Supplementary figure 8.** Frontal cortex section scores.


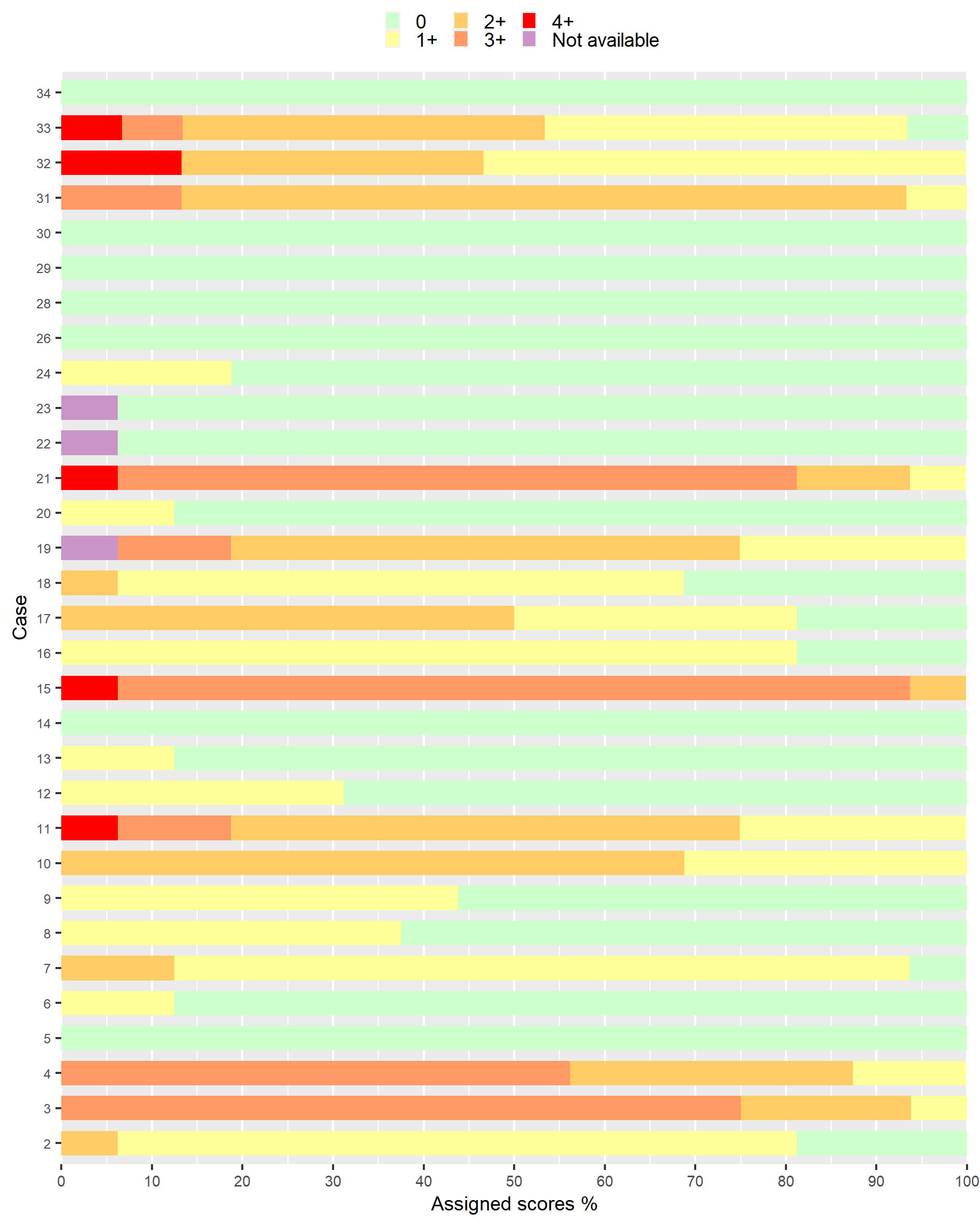


**Supplementary figure 9.** Parietal cortex section scores.


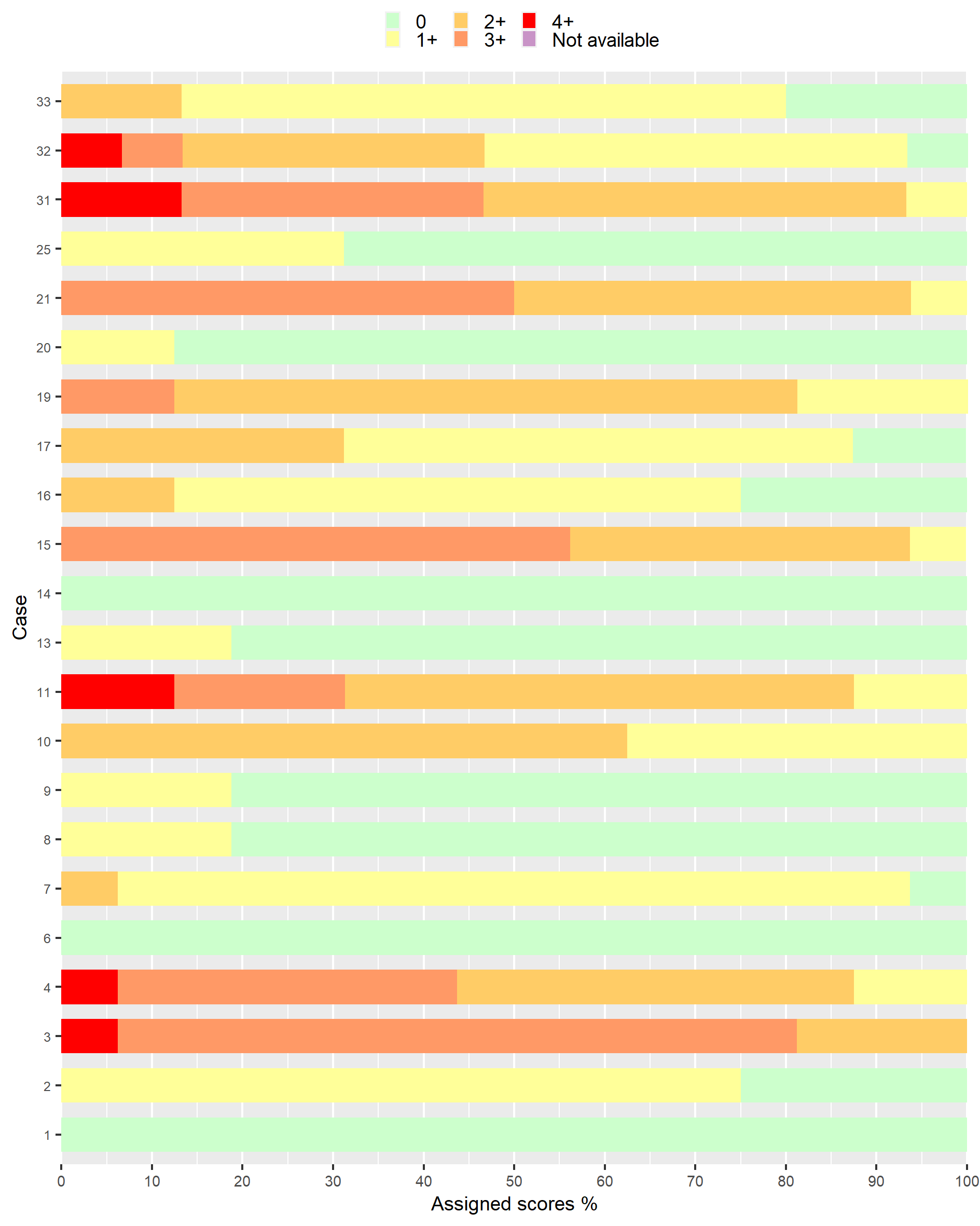

Supplement: Supplementary file 1 — Supplementary file1 (DOCX 6898 KB) [file 401_2020_2255_MOESM1_ESM.docx]
